# Vitamin—Conjugated Metallic Nanoparticles: Applications for Antimicrobial and Anti-Cancer Drug Delivery

**DOI:** 10.3390/molecules30214248

**Published:** 2025-10-31

**Authors:** Meriama Genamo, Addisie Geremew, Elisha Peace, Laura Carson

**Affiliations:** 1 Department of Chemistry, College of Arts and Sciences, Prairie View A&M University, Prairie View, TX 77446, USA; mgenamo@pvamu.edu; 2 Cooperative Agriculture Research Center, College of Agriculture, Food and Natural Resources, Prairie View A&M University, MS 2008, P.O. Box 519, Prairie View, TX 77446, USA; aygeremew@pvamu.edu (A.G.); efpeace@pvamu.edu (E.P.)

**Keywords:** targeted drug delivery, antimicrobial therapy, anticancer therapy, surface functionalization, cellular uptake, drug resistance

## Abstract

Vitamin-conjugated metallic nanoparticles (VC-MNPs) have emerged as a transformative platform in nanomedicine that combine the therapeutic potential of vitamins with the structural versatility of metal nanoparticles. They offer a dual advantage of targeted drug delivery and enhanced therapeutic efficacy, enabling precise intervention against infectious and malignant diseases. Vitamin conjugation facilitates receptor-mediated targeting, antioxidant enhancement, and improved biocompatibility, thereby strengthening therapeutic outcomes and reducing off-target effects. This review critically evaluates how vitamin functionalization modulates the synthesis, activity, and clinical translation of VC-MNPs. Diverse synthesis methods including chemical reduction, co-precipitation, sol–gel, and green approaches are evaluated, along with the influence of synthesis parameters on nanoparticle performance. The mechanisms underlying enhanced antimicrobial and anti-cancer efficacy are discussed, highlighting the contributions of vitamin functionalization to cellular uptake, redox balance and metabolic selectivity. Critical challenges in clinical translation are systematically assessed, including nanoparticle stability under physiological conditions, potential toxicity concerns, regulatory approval pathways, and manufacturing scalability requirements. Finally, the paper considers future perspectives, focusing on synthesis innovations, novel therapeutic targets, interdisciplinary collaborations, and pathways for clinical translation. Overall, VC-MNPs represent a promising next-generation platform for precision nanomedicine and sustainable therapeutic design.

## 1. Introduction

### Overview of Nanotechnology and Its Significance in Drug Delivery

**Figure 1:**
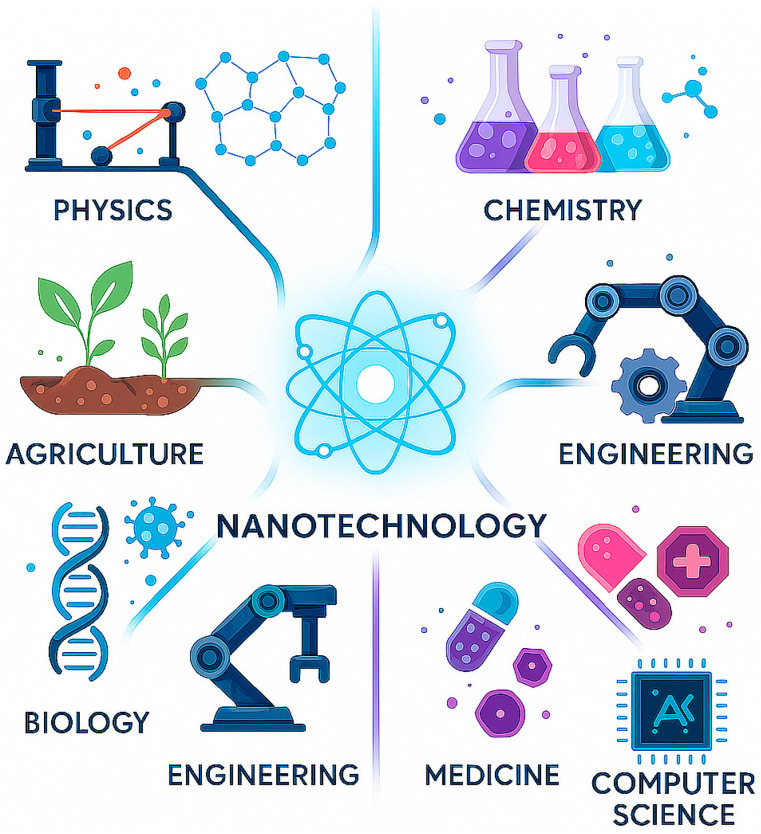
Application of nanotechnology in different fields. This image was generated using Chat GPT 5.

Recent advancements in nanotechnology have significantly influenced a broad spectrum of scientific disciplines encompassing physics, materials science, chemistry, biology, computer science, medicine, and engineering (Figure 1). Increasingly, research efforts are directed toward the precise control of atomic and molecular arrangements at the nanoscale to engineer structures, devices, and systems with novel functionalities and enhanced performance characteristics [1]. This broad impact is perhaps most evident in medicine, where nanotechnology has emerged as a transformative paradigm in drug delivery; revolutionizing how therapies are delivered at the cellular level [2,3]. Characterized by the manipulation of materials at the nanometer scale (1–100 nm), nanoparticles (NPs) possess distinctive physicochemical attributes such as elevated surface area-to-volume ratios, customizable surface functionalities, and enhanced permeability that render them highly suitable for targeted therapeutic interventions [4]. Consequently, these unique features enable NPs to circumvent many of the inherent limitations associated with conventional drug delivery systems, including poor solubility, limited bioavailability, and system toxicity [5].

NPs can be fabricated from a diverse array of materials whose physicochemical properties can be engineered based on specific clinical objectives including drug delivery, imaging, or stimulus-responsive therapy. Commonly employed materials including organic polymers such as (e.g., chitosan and PLGA) [6], lipids (e.g., phospholipids, PEG, and nucleic acids) [7] and inorganic cores such as metals (e.g., Au, Ag, Fe, Zn, Cu) or silica [8,9]. Lipid and polymeric NPs are superior at encapsulating hydrophobic drugs such as doxorubicin and paclitaxel improving solubility, delivery, and reducing off-target toxicity [10]. Each class of material offers distinct properties that are particularly tailored to specific therapeutic and diagnostic applications. In contrast, metallic nanoparticles (MNPs) offer high surface area, tunable surface chemistry, and unique optical, electronic, and magnetic properties that make them especially attractive for imaging, photothermal therapy, and multifunctional theranostics [11,12].

Effective drug delivery remains a cornerstone of modern therapeutics, ensuring optimal pharmacological outcomes while minimizing adverse effects [13]. In this regard, nanoparticle-based delivery systems have demonstrated considerable promise across various medical disciplines, including oncology, neurology, infectious diseases, and cardiovascular medicine. For example, in oncology, targeted lipid and polymeric NPs selectively accumulate in tumors, enhancing the delivery of drugs like doxorubicin and paclitaxel in breast and lung cancers while minimizing cardiotoxicity and systemic toxicity which improve therapeutic efficacy [14,15,16]. In neurological disorders, engineered NPs can cross the blood–brain barrier, overcoming a major limitation of conventional therapeutics [17]. Similar strategies implement in cardiovascular medicine, where NPs can localize thrombolytic or anti-inflammatory agents to diseased tissues and reduce systemic risks [18]. In contrast to conventional delivery methods prone to instability, rapid clearance, and poor selectivity, targeted NPs delivery maximizes drugs accumulation at disease sites while reducing systemic toxicity [19,20]. Moreover, emerging strategies such as exosome-derived NPs and green synthesis approaches further improve biocompatibility and sustainability [21].

The NPs tunable physicochemical parameters such as size, shape, composition, and surface functionalization, enable receptor-mediated uptake via conjugation with ligands, antibodies, or polymers, and facilitate stimuli-responsive release triggered by pH, redox, or temperature gradients [22,23].

Phytochemicals have emerged as key agents in the green synthesis of NPs, owing biocompatibility and wide spectrum of therapeutic benefits including antimicrobial and anticancer activities [24]. Among these phytoconstituents, vitamins and essential micronutrients show defined physiological roles and have gained high attention for their multiple therapeutic domains such as antioxidant, anticancer, antimicrobial, and antidiabetic properties [25,26].

Despite these promising attributes, their conventional administration as clinical utility is hindered by inherent challenges, of chemical instability, rapid metabolic degradation, and limited bioavailability [27]. To address the limitations, nanotechnology has promoted the transformative strategies in which vitamins are conjugated with NPs, to enhance pharmacokinetic profiles and enabling targeted delivery where the vitamin promotes cellular uptake and the NPs core enhances drug delivery [28,29].

Focusing on metallic nanocarriers, vitamin-conjugated metallic nanoparticles (VC-MNPs) have gained particular attention as multifunctional therapeutic systems. Vitamins such as folate, biotin, B-complexes, vitamins C or D can coordinate to metal surfaces, enabling stable functionalization and receptor targeting [30]. For instance, folate-conjugated MNPs exploit folate receptor overexpression in many cancers, improving tumor selectivity [31]. Biotin has also been used to facilitate targeted delivery due to its high affinity for avidin/streptavidin systems [32], while thiamine (B1) and cobalamin (B12) stabilize MNPs via heteroatom coordination and redox-mediated metal reduction, respectively [33]. Beyond MNPs, vitamin functionalized liposomal, lipid-based, and polymeric nanocarriers have demonstrated superior therapeutic efficacy and safety outperforms the traditional formulations [34,35,36,37].

Despite these advances, existing literature often remains descriptive, emphasizing general properties and synthesis methods of metallic nanoparticles rather than their biofunctionalization; a critical factor influencing biological performance [7]. In this context, vitamin conjugation represents a distinctive and underexplored strategy for targeted delivery, biocompatibility enhancement, and dual therapeutic ligand activity. Therefore, this review critically discusses VC-MNPs as a unique hybrid nanoplatform that unites the physicochemical strengths of MNPs with the biological advantages of vitamins. It further examines recent advances in synthesis, characterization, and biomedical applications, emphasizing their therapeutic potential and providing detailed insights into the mechanisms of action.

## 2. Methods of Metal Nanoparticles Synthesis

Nanomaterial synthesis typically follows either a top-down approach: where bulk materials are reduced to nanoscale via techniques such as milling, sputtering, grinding, and thermal treatment or a bottom-up approach: which utilizes chemical and biological methods to assemble NPs atom by atom. This review emphasizes various solution-based synthesis strategies which promote functionalization and conjugation of biologically active molecules on to NPs. These method is carried out under mild, biocompatible and controllable conditions aimed to enhance targeting and therapeutic efficacy of fabricated nanomaterials [38,39,40].

### 2.1. Chemical Approach

Chemical synthesis of MNPs involves three primary components: metal salt precursors, reducing agents, and capping/stabilizing agents that help to coat the surface, prevent aggregation and control morphology. Common chemical techniques include chemical reduction [41], co-precipitation [42], and sol–gel methods, each provides distinct advantages and limitations depending on the intended applications [43]. In chemical reduction, reducing agents such as borohydrides, citric/oxalic acids, polyols, hydrogen peroxide, and sulfites facilitate electron transfers to metal ions, forming free atoms. Additionally, stabilizing agents like trisodium citrate dihydrate, sulfur ligands (thiolates), phosphorus ligands, polymers, and surfactants (e.g., cetyltrimethylammonium bromide, CTAB) are employed to prevent nanoparticle aggregation and improve uniformity and dispersion [44,45,46]. Although the chemical approach offers precise control over size and shape the toxicity of reductants such as sodium borohydride, hydrazine poses serious health and environmental risks including neurotoxicity and ecological contamination [47,48,49,50].

The co-precipitation method involves simultaneous precipitation of multiple metal ions like as Fe^2+^/Fe^3+^ in alkaline media using NaOH or NH_4_OH to produce uniformly sized NPs with controlled composition and properties. Even though, the method allows rapid, large-scale synthesis of magnetite and other oxide NPs under mild conditions; it often leads to particle aggregation and poor control over oxidation states, which requires surfactant additives such as CTAB, cetyltrimethylammonium chloride (CTAC), or PEG to enhance stability [51,52,53].

The sol–gel process encompasses hydrolysis, polymerization/condensation of monomers, particle growth, and gel formation. It involves the phase transformation of a colloidal “sol” into a solid “gel.” Its mild processing temperature supports the inclusion of organic or bioactive moieties, making it particularly suitable for hybrid materials used in drug delivery and biosensing. Despite its precision, sol–gel synthesis often suffers from extended reaction times and costly precursors [54,55,56,57].

Although, chemical synthesis ensures reproducibility and fine control over nanoparticle characteristics, it suffers from poor biocompatibility and toxic byproducts which limits its direct biomedical applications. Therefore, this has driven a transition toward vitamin-conjugated or biologically functionalized MNPs that integrate the structural precision of chemical synthesis with enhanced safety and bioactivity.

### 2.2. Greener Approach

**Figure 2:**
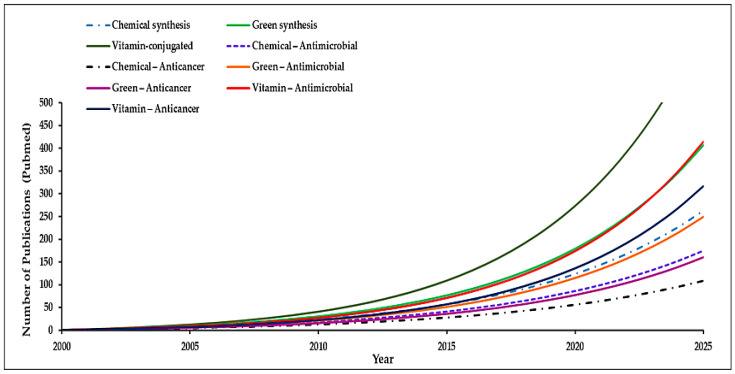
Trend analysis of synthesis techniques used for metallic nanoparticles from 2000 to min 2025.

Recently, environmentally sustained synthesis of nanomaterials shows an increasing trend (Figure 2) and has gained significant attention due to its effectiveness, low cost, reduced failure rates, and ease of characterization, which makes it preferable over conventional methods [58]. This ecofriendly approach uses bioactive compounds from natural sources such as enzymes, vitamins, carbohydrates, bacteria, fungi, yeast, algae, and plant secondary metabolites which act simultaneously as capping and reducing agents. Such biologically synthesized NPs exhibit superior biocompatibility, nano-dimensions, and pharmacological properties, making them suitable for biomedical applications such as drug delivery, cancer therapy, antimicrobial treatments, and tissue regeneration [59].

Among biological mediators, vitamins play vital role owing to their redox-active functional groups such as hydroxyl, amine, and carboxyl moieties and their inherent therapeutic values. The conjugation of vitamins such as folic acid, riboflavin, thiamine, biotin, and vitamins C and D on to metallic nanoparticle surfaces has been shown to improve stability, solubility, and targeted bio-interactions (Table 1). For instance, Singh et al. [60] demonstrated that riboflavin and thiamine functionalized carbon nanotubes exhibit potent cytotoxicity against MCF-7 breast cancer cells. Chakraborty and Jana [61] also reported vitamin C-conjugated gold nanoparticles (AuNPs) with superior oxidative stress attenuation ability compared to free vitamin C. Similarly, vitamin D-conjugated AuNPs promotes osteogenic differentiation in human adipose-derived stem cells (hADSCs) [62]. Chemically synthesized VC-MNPs often rely on post-synthetic functionalization including mixing pre-formed NPs with vitamin solutions under optimized pH and temperature to achieve surface conjugation and maintain structural integrity [63,64]. In contrast, green methods integrate vitamin molecules directly during the reduction phase, when they serve simultaneously as nucleation initiators and capping agents. This intrinsic coupling generally results in better surface passivation and biocompatibility. VC-MNPs thus represent a compelling hybrid paradigm, merging synthetic precision with biological efficacy, poised to bridge the gap between engineered control and living compatibility [65].

Despite remarkable advances, scalability and reproducibility remain the primary bottlenecks for green synthesis. Variability in biological raw materials across season, uncontrolled reaction kinetics, and limited mechanistic understanding hinder consistent translation to industrial scale. To overcome these barriers, investigating vitamin mediated reduction and metal bindings at molecular level, and incorporating computational modeling and machine learning to optimize reaction parameters for predictable and reproducible outcomes is valuable [66].

**Table 1:** Summary of metal nanoparticles with vitamins.

S/N	Metal NP + Vitamin	Synthesis Method	Size & Morphology	Characterization Techniques	Applications	Key Findings	Ref.
1	Ag (Ag/Cu) + Ascorbic Acid	Chemical reduction	Ag: ~200–800 nm; Cu: ~160–630 nm spherical	UV–Vis, DLS, TEM	Antibacterial: Tested against *Bacillus subtilis* (Gram+) and *E. coli* (Gram−).	Strongest bactericidal effect (MIC ~0.05–0.08 mg/L).	[67]
2	Au + Vitamin C (with algal EPS)	Green biosynthesis	~6–40 nm spherical AuNPs	UV–Vis, XRD, TEM, FTIR	Antibacterial: Multi-strain (*E. coli*, *S. aureus*, *S. enterica*, *S. mutans*, *Candida* spp.). Anticancer: Tested against MCF-7, A549, and CaCo-2 cells.	Most effective: >88% kill of *E. coli* and ~83% of *S. aureus* under light (via ROS generation), and ~70% growth inhibition of MCF-7 breast cancer cells.	[68]
3	CeO_2_ + Folic Acid	Green one-pot precipitation	~21–28 nm polyhedral; DLS (hydrodynamic ~200 nm; 22 mV)	XRD, TEM/SEM	Antibacterial: Potent against MRSA. Antioxidant/Anti-inflammatory. Anticancer (MDA-MB-231).	Inhibit ~95.6% of MRSA growth, accelerated wound healing and selective toxicity toward bacteria and cancer cells.	[69]
4	Cu–MOF + Folic Acid (Cu-TCPP MOF/Pt-FA)	Chemical method	Nanosheets ~100–200 nm; Pt NPs ~2 nm	TEM/HRTEM, XRD, XPS, FTIR, Zeta potential	Anticancer (PDT & immunotherapy)	Greatly enhances PDT even in hypoxic conditions.	[70]
5	Cu_2_S + Vitamin C	Single-step aqueous synthesis (Chemical reduction)	CuS ~8–10 nm, quasi-spherical; agglomerates into 50–100 nm clusters	XRD, FTIR, TEM/SEM, EDS	Antibacterial (*S. aureus*, *E. coli*, *K. pneumoniae*), Antioxidant	Broad-spectrum bactericidal activity. MIC: ~2 mg/mL (*E. coli*) and 10 µg/mL (other strains). Also scavenged DPPH & NO radicals.	[71]
6	Fe_3_O_4_ + Riboflavin (B_2_)	Solvothermal method	~200 nm spherical	XRD, XPS, Zeta potential, UV-Vis, Fluorescence	Antibacterial and Antioxidant	Kills >90% of *S. aureus* and ~88% of *E. coli* at 0.5 mg/mL.	[72]
7	Fe_3_O_4_ + Folic Acid (PLGA nanocarrier)	Double emulsion solvent evaporation	~150–180 nm polymeric spheres (PLGA); Fe_3_O_4_ cores ~8 nm	TEM, DLS, FTIR, NMR, MRI	Anticancer	Induce ~90% cell death in ovarian cancer.	[73]
8	Fe_3_O_4_ + Folic/TNF/IFN/DOX	Surface functionalization & self-assembly	Fe_3_O_4_ core ~5 nm; clusters ~50 nm spherical	FTIR, DLS, UV–Vis	Anticancer (combined therapy)	Produced synergistic cancer cell killing with reduced systemic toxicity.	
9	Ag + Folic Acid (valve coating)	Biofunctional coating	~10 nm AgNPs	SEM, EDS, FTIR	Antibacterial & anti-inflammatory implant	Reduced calcification and inflammation in vivo.	[74]
10	Ag/MOF + Folic Acid (nanocapsule)	Biopolymer-templated in situ MOF synthesis	~320–350 nm mixture of rod-like and spherical particles	XRD, FTIR, SEM, TEM, BET	Antibacterial, antioxidant, targeted drug delivery	Single folate-targeted nanocapsule can deliver chemotherapeutics while preventing infection & oxidative damage.	[75]
11	Gd_2_O_3_ + Vitamin C	Biogenic precipitation	~50 nm amorphous Gd_2_O_3_ particles	TEM, DLS, XPS, ICP	Antibacterial	Potent bactericidal effects against multiple pathogens.	[76]
12	Au + Riboflavin (B_2_)	Photochemical surface-modification	AuNP ~20 nm, spherical	UV–Vis	Photodynamic antimicrobial therapy (*S. aureus*, *P. aeruginosa*)	Vitamin B_2_ + AuNP create synergistic ROS + Au^+^ antibacterial effect.	[77]
13	Ag + α-Tocopherol Succinate (Vit E)	Surface functionalization	Ag core ~20 nm; hydrodynamic size ~25 nm (TOS coating)	UV–Vis, FTIR, DLS/Zeta potential	Anticancer (A549 lung carcinoma)	TOS coating enhanced cancer selectivity & therapeutic index of AgNPs.	[78]
14	Y_2_O_3_ + Folic Acid	Chemical synthesis/thermal decomposition	~5–10 nm hexagonal phase; aggregates into ~100 nm clusters; folate-PEG ~120 nm	Photoluminescence, TEM, FTIR, DLS	Cancer imaging	Enabled precise NIR-triggered imaging & potential phototherapy.	[79]
15	Se + Vitamin C	Chemical reduction	~50–60 nm spherical	UV–Vis, DLS, Zeta potential, XPS	Antibacterial	Strong activity against *S. aureus*; stabilized Se–VitC NPs retained activity 2–6 months.	[80]
16	Zn/Ag MOF + Vitamin C	Chemical synthesis with functionalization	~100–200 nm polyhedral	XRD, TEM, SEM, FTIR, BET	Antibacterial	Strong activity against Gram+ and Gram− bacteria common in wound infections.	[81]
17	Fe/MOF + Riboflavin	Hydrothermal method	Uniform polyhedral morphology	TEM, DLS, Zeta potential, XRD, FTIR, SEM, EDS, UV-Vis, Thermal imaging	Treatment of bacterial keratitis (*S. aureus*, *P. aeruginosa*)	Rapid infection clearance with minimal collateral damage.	[82]
18	Ag NPs + Biotin, D-Pantothenic acid & Nicotinic acid	Chemical reduction with NaBH_4_	~10 nm spherical	UV-Vis, TEM, FTIR, DLS, TGA, FE-STEM	Antimicrobial	Effective at low concentrations (15.62–62.5 μg/mL) against planktonic cells & biofilms.	[83]

### 2.3. Influence of Synthesis Parameters

The physicochemical characteristics of materials, including their composition and morphology, are profoundly influenced by various synthesis parameters. Among these pH, reactant concentration, reaction duration, and temperature are the major factors, each of which can be strategically manipulated to tailor material properties [84,85]. Specifically, temperature and pH play pivotal roles in determining the size, shape, and formation rate of NPs. Elevated pH levels have been shown to enhance the nucleation process, thereby accelerating the reduction in metal ions into MNPs [86]. Building on this, Fernando and Zhou [87] reported that AgNPs exhibited improved stability and decreased dissolution at pH ≥ 9, while neutral or acidic pH induced aggregation and oxidative degradation. Similarly, Awadh et al. [88] found that ZnO NPs synthesized at pH 10–11 displayed well-defined UV–Vis absorption peaks (364–366 nm), whereas excessive alkalinity (pH ≥ 12) led to particle aging and broad size distributions. Temperature also played a crucial role; overheating can destabilize organic ligands and induce morphological deformation. Moderate temperatures (60–80 °C) often yield narrow size distributions in Au and Ag systems, while temperatures above 90 °C cause ligand decomposition or sintering [89,90,91]. This sensitivity to pH and temperature is not limited to synthesis but extends to biomedical applications, where these parameters critically affect drug loading and release in nanocarrier systems. Recently, pH-responsive nanodrug delivery platforms have garnered considerable interest for their ability to selectively deliver therapeutic and diagnostic agents to diseased tissues. The pH of the target environment influences both the binding efficiency of functional ligands on the nanoparticle surface and the release kinetics of the encapsulated drug as improper pH control may lead to off-target effects and toxicity in healthy tissues [92,93]. In addition to pH and temperature, both reactant concentration and reaction duration are crucial in controlling size and growth of NPs during synthesis. Higher precursor concentration increases nucleation frequency but risks uncontrolled aggregation, while prolonged reaction times lead to Ostwald ripening and size broadening [94,95,96]. For example, Dehsari et al. [97] reported that the effect of precursor concentration on the size of iron oxide NPs, showing that increasing Fe^3+^ concentration reduced Fe_3_O_4_ nanoparticle size up to an optimal threshold, beyond the threshold point excessive ion availability promoted particle agglomeration.

## 3. Characterization Techniques of Nanoparticles

Characterizing NPs behavior in biomedical applications necessitates comprehensive evaluation of their fundamental properties. Critical determinants such as particle dimensionality, morphological architecture, surface electrostatics, porosity, polydispersity index, and specific surface area significantly influence their interactive dynamics and efficacy in targeted applications, encompassing drug delivery systems, catalytic processes, biomedical imaging, and environmental detoxification [98,99]. Comprehensive and precise characterization of these parameters is imperative for elucidating NPs behavior and refining synthesis methods to enhance application-specific performance. In VC-MNPs, these factors critically determine the stability of conjugation, antioxidant efficiency, and the effectiveness of receptor-mediated targeting [100].

To achieve this, a multifaceted suite of analytical methods is employed, each offering distinct and complementary insights into nanoparticle composition and functionality. These techniques encompass Transmission Electron Microscopy (TEM), Scanning Electron Microscopy (SEM), Ultraviolet–Visible (UV-Vis) Spectroscopy, Fourier Transform Infrared (FTIR) Spectroscopy, X-ray Diffraction (XRD), X-ray Photoelectron Spectroscopy (XPS), Dynamic Light Scattering (DLS), Atomic Force Microscopy (AFM), Nuclear Magnetic Resonance (NMR) Spectroscopy, and zeta potential analysis. Collectively, these tools facilitate a holistic understanding of NPs physicochemical profiles (Table 1), thereby informing their rational design and application [101].

### 3.1. Morphological and Structural Characterization

Electron microscopy techniques serve as essential analytical tools for nanoparticle structural characterization. TEM facilitates ultra-high-resolution imaging, enabling the direct visualization of internal nanostructures and precise morphological delineation at the atomic scale, which is critical for examining spatial distribution and interfacial architecture. In parallel, SEM complements this analysis by generating three-dimensional surface topographies through secondary electron detection, thereby offering critical insights into surface morphology, texture, and particulate arrangement [102]. However, while TEM offers exceptionally nanoscale resolution, it may induce beam-damage artifacts, and SEM requires conductive coatings that may obscure organic layers such as vitamins [103,104]. Consequently, combining these methods with non-destructive techniques such as AFM enables a more realistic visualization of the hydrated VC-MNPs surface [105].

In addition to morphological elucidation, crystallographic and molecular-level insights are also useful for verifying the structural integrity of VC-MNPs. XRD serves as a definitive tool for crystallographic analysis, offering quantitative data for metal-ligand conjugates on lattice parameters, crystallite dimensions, and phase composition. However, XRD cannot resolve amorphous organic coatings and complementary techniques such as TEM, DLS or XPS are required to provides a holistic understanding of nanoparticle architecture from atomic arrangement to colloidal behavior [100,106].

### 3.2. Optical and Spectroscopic Characterization

Beyond morphological characterization, UV-Vis spectroscopy introduces a rapid, non-invasive optical modality for monitoring nanoparticle synthesis and colloidal stability. Particularly efficacious for metallic NPs due to their surface plasmon resonance (SPR) phenomena, UV-Vis spectral profiles specifically the peak position and bandwidth serve as proxies for particle size, dispersion uniformity, and aggregation dynamics, linking structural and optical properties in real time [107]. Nevertheless, UV–Vis data alone are insufficient to confirm covalent conjugation and must be validated by FTIR spectroscopy, which identifies functional groups involved in metal coordination [108]. But, due to overlapping peaks from metal–ligand or vitamin–protein interactions it results in complicated interpretation. In this scenario, NMR spectroscopy provides molecular level resolution of ligand–metal binding environments, although its sensitivity is limited for heterogeneous nanocomposites [109].

### 3.3. Surface Characterization and Stability Profiling

Surface characterization is a crucial in understanding the performance and biocompatibility of biomedical nanomaterials [110]. XPS introduces a surface-sensitive analytical dimension, capable of resolving elemental composition and oxidation states within the top few nanometers of the nanoparticle surface. This technique is critical for validating surface modifications, dopant incorporation, and functional group presence, thereby linking surface chemistry to electronic structure and catalytic potential [111].

DLS serves as a pivotal analytical technique for characterizing colloidal systems by determining the hydrodynamic diameter and polydispersity index, thereby offering insights into particle size distribution and uniformity. When coupled with zeta potential analysis, DLS extends its utility to evaluating the electrostatic potential at the nanoparticle interface, a critical parameter for assessing colloidal stability. High absolute values of zeta potential regardless of polarity signify strong interparticle repulsion, which effectively minimizes aggregation and enhances dispersion stability under varying environmental conditions. This dual capability is instrumental in predicting nanoparticle behavior in both biological and industrial contexts, particularly with respect to long-term storage and functional performance. Moreover, DLS complements spectroscopic techniques such as FTIR by bridging surface chemistry with physicochemical stability, thus providing a comprehensive framework for nanoparticle evaluation [112,113]. However, since DLS measures average sizes across hydration layers and cannot distinguish between the nanoparticle core and surface corona, it requires correlation with TEM data for accurate interpretation.

## 4. Applications

### 4.1. Antimicrobial Applications

The rise in multidrug-resistant (MDR) pathogens has intensified the search for alternative antimicrobial strategies. Among MNPs such as silver, platinum, zinc oxide, and copper have shown potent antimicrobial properties. Functionalizing these NPs with vitamins (Table 1) further enhances their efficacy, selectivity, and safety profile and also improves their stability, solubility, and bioavailability [114]. These NPs exhibit strong efficacy against both bacterial and fungal pathogens, often outperforming conventional antibiotics [115,116].

Importantly, VC-MNPs have been reported to improve antimicrobial activity when incorporated into drug delivery systems, resulting in enhanced pharmacokinetics, reduced dosage requirements, and minimized toxicity [117]. These properties position them as promising candidates for the treatment of persistent and resistant infections, with ongoing research demonstrating synergistic effects and broadened antimicrobial spectra when combined with commercial antimicrobial agents [118,119]. Additionally, these NPs are increasingly incorporated into coatings for surgical instruments, wound dressings, and implantable devices to prevent microbial colonization and biofilm formation, thereby reducing infection rates in clinical settings [120].

### 4.2. Anti-Cancer Applications

Vitamin-functionalized metallic nanoparticles have emerged as versatile platforms for cancer therapy, integrating the diagnostic precision of metal nanostructures with the selective targeting capabilities of vitamin ligands [121]. Several case studies highlight that specific nanoparticle formulations, such as Au or Pt conjugated with vitamins, can enhance immunotherapeutic responses, promote targeted drug delivery, and induce tumor cell death through mechanisms like immunogenic cell death and photothermal/photodynamic therapy [122,123]. Combination therapies that integrate these NPs with existing treatments have shown additive or even synergistic effects, often resulting in improved tumor suppression in preclinical models. Some have advanced to early-phase clinical evaluation, where the focus has been on enhancing efficacy while minimizing off-target toxicity [124,125,126]. For example, folate-conjugated Au and Gd NPs demonstrated enhanced accumulation in folate-receptor positive tumors compared with non-targeted PEG-AuNPs, resulting in reduced off target toxicity [127,128]. Similarly, riboflavin-AgNPs exhibit superior apoptosis induction compared with free drugs due to synergistic oxidative and photothermal effects [129]. Unlike traditional delivery vehicles such as liposomes or polymeric micelles, VC-MNPs offer integrated therapeutic and diagnostic functions, enabling simultaneous drug delivery and real-time imaging through computed tomography (CT), magnetic resonance imaging (MRI), or fluorescence modalities. Their tunable optical and redox properties further support photothermal and photodynamic treatments. Notably, formulations like folate-AuNPs and biotin-PtNPs have advanced to early clinical trials for targeted chemotherapy and imaging applications [127,130].

### 4.3. Other Emerging Applications

Beyond antimicrobial and anticancer therapeutic uses, VC-MNPs are gaining traction in diagnostic imaging, gene delivery, and biosensor technologies. Compared with traditional fluorescent or enzyme-based biosensors, vitamin-functionalized nanometals exhibit enhanced selectivity due to ligand–receptor affinity and catalytic amplification capabilities [131,132]. For instance, biotinylated NPs are being explored in biosensors for their ability to selectively bind to target biomolecules, enabling rapid and accurate detection of pathogens or disease biomarkers [133,134]. In diagnostic imaging, VC-MNPs enhance the specificity and sensitivity of imaging modalities such as MRI, CT, and PET by targeting overexpressed vitamin receptors on diseased cells [135]. Folic acid–AuNPs and riboflavin–Fe_3_O_4_ NPs have demonstrated superior MRI and CT contrast in tumor imaging relative to commercial gadolinium-based agents [136,137]. Similarly, vitamin D-conjugated Au NPs have been shown to enhance osteogenic differentiation in stem cells, indicating potential applications in bone regeneration and tissue engineering [62].

In gene delivery systems, VC-MNPs offer a promising non-viral alternative for transporting genetic material into cells. These platforms have demonstrated versatility in delivering a range of genetic payloads, such as siRNA for gene silencing, plasmids for gene expression, and CRISPR-Cas9 components for genome editing, thereby enabling precise genetic modulation for various therapeutic purposes [138,139]. As a result, their application extends to the treatment of inherited disorders, cancer, and other diseases where targeted gene regulation is crucial [140,141]. Clinical translation efforts are now focusing on the development and approval of non-viral gene therapy products, with several clinical trials underway for conditions such as genetic disorders and cancers, highlighting the growing recognition of VC-MNPs as safer and more versatile alternatives [140,142].

## 5. Mechanisms of Actions

Deciphering the mechanisms through which pharmacological compounds exert their therapeutic effects is crucial for advancing drug development and optimizing therapeutic interventions. The mechanism of action refers to how the drug works in the body by interacting with a specific target such as enzymes, ion channels, or membrane-bound receptors to produce intended therapeutic response. These interactions dictate drug efficacy, selectivity, and safety by modulating intracellular signaling, enzyme activity, or receptor-mediated processes [143,144]. Drug efficacy is frequently mediated through competitive inhibition, wherein the therapeutic agent competes with endogenous substrates or ligands to modulate enzymatic activity or receptor-mediated signaling pathways. Through this mechanism, drugs can either mimic the actions of natural ligands as agonists or block receptor activation as antagonists, ultimately determining the cellular response and therapeutic outcome [145]. In redox-regulated pathways, therapeutic compounds can help establish cellular homeostasis by scavenging reactive oxygen species (ROS), modulating redox sensitive transcription factors such as Nuclear factor erythroid 2-related factor 2 (NRF2), or enhancing endogenous antioxidant enzymes such as superoxide dismutase, catalase, and glutathione peroxidase [146]. Disruption of this balance between oxidants and antioxidants contributes to disease progression, underscoring the therapeutic relevance of redox active systems [147,148,149]. Elucidating these molecular processes enables rational design of multifunctional therapeutics particularly VC-MNPs, which integrate biochemical targeting with enhanced cellular selectivity [150]. The following sections discuss the effect of vitamin functionalization on mechanism of action of antimicrobial and anticancer agents.

### 5.1. Antimicrobial Mechanisms

#### 5.1.1. Inhibition of Bacterial Growth

Dissecting antimicrobial mechanisms at the molecular and genetic level provides critical insights into how VC-MNPs disrupt bacterial physiology and overcome resistance mechanisms [151]. VC-MNPs demonstrate potent antimicrobial activity through multiple interconnected mechanisms that synergistically inhibit bacterial growth [152]. The unique physicochemical features of NPs particularly their high surface-to-volume ratio and nanoscale dimensions (1–100 nm) facilitate strong interactions with bacterial membranes, enabling efficient penetration and cooperative modulation of cellular processes through the combined effects of the metal core and vitamin ligands [153,154,155]. At the molecular level, the antimicrobial effects involve comprehensive disruption of bacterial gene expression patterns and cellular processes. Transcriptomic analyses have revealed that nanoparticle treatment alters fundamental bacterial processes including protein translation, energy metabolism, and stress response pathways [156].

**Figure 3:**
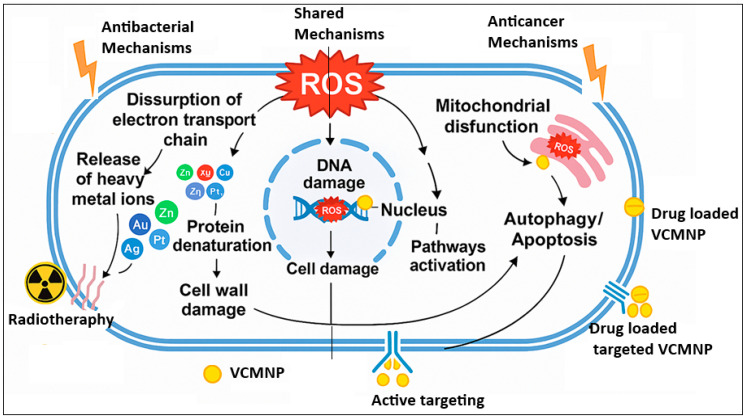
Antibacterial and anticancer mechanisms of VC-MNPs. Shared mechanisms connected in straight line without arrow. The image was generated using Microsoft 365 Copilot.

These broad transcriptional changes occur before bacterial population density changes, indicating that NPs directly interfere with essential gene regulatory networks rather than simply reducing bacterial numbers [157]. The antimicrobial activity of vitamin-conjugated NPs operates through both contact killing where electrostatic and hydrophobic interactions cause membrane rupture and leakage of cellular contents and ion-mediated killing mechanisms in which involving sustained release of metal ions that bind sulfhydryl groups in enzymes, inactivating key metabolic proteins and impairing ATP synthesis [158,159,160]. Furthermore, VC-MNPs can produce reactive oxygen species such as hydroxyl radicals, superoxide anions, and hydrogen peroxide, which trigger oxidative stress within bacterial cells [161]. This results in damage to vital biomolecules (Figure 3), including lipids, proteins, and DNA and culminates in apoptosis-like cell death [61,161]. The damage in bacterial DNA causes mutation and disruption of critical genetic processes such as replication and transcription [154]. The antibacterial mechanisms vary between Gram-positive and Gram-negative bacteria due to differences in their cell envelope structures. Gram-negative bacteria possess an outer lipopolysaccharide layer that restricts nanoparticle entry, while the thick peptidoglycan wall of Gram-positive bacteria enables stronger electrostatic binding with cationic NPs, resulting in greater membrane damage and oxidative stress. These structural distinctions govern bacterial susceptibility and influence the overall efficacy of VC-MNP antibacterial activity [162].

#### 5.1.2. Mechanisms of Resistance Overcoming

Unlike single-target antibiotics, VC-MNPs attack multiple bacterial pathways simultaneously, making the development of stable resistance improbable [163,164]. From a genetic standpoint, resistance mechanisms in bacteria involve specific genes that encode proteins responsible for antibiotic degradation, efflux pumps, and target modification. They interfere with genetic resistance determinants including thioredoxin A (trxA), thioredoxin reductase (trxB), D-alanine-poly phosphoribitol (dltA), and efflux pump genes multidrug efflux membrane fusion proteins (mexA–mexC) through DNA binding and oxidative disruption, impairing transcriptional responses [165,166]. Also, biofilm penetration represents a significant advantage of vitamin-conjugated NPs in overcoming resistance [167]. Many antibiotic-resistant bacteria exist within biofilms that provide protection against conventional treatments. The small size and surface properties of vitamin-conjugated nanoparticles enable them to penetrate these biofilm matrices and reach embedded bacterial cells [154,167]. A major advantage of VC-MNPs lies in their capacity to penetrate and disrupt microbial biofilms, a major barrier in chronic infections [167]. VC-MNPs disrupt quorum-sensing genes like as autoinducer-2 synthase (luxS), N-3-oxo-dodecanoyl-homoserine lactone synthase (lasI), N-butanoyl-homoserine lactone transcriptional regulator (rhlR) and adhesion regulators such as fimbrial adhesion subunit (fimH), Biofilm-associated protein (bap); preventing biofilm maturation and dismantling established matrices [166,168].

Moreover, the vitamin targeting component provides specificity that helps overcome resistance by exploiting bacterial vitamin requirements. Bacteria require various vitamins for essential metabolic processes, and vitamin-conjugated NPs can hijack these uptake pathways to deliver antimicrobial agents directly into resistant bacterial cells. This targeted approach bypasses many traditional resistance mechanisms that bacteria use to exclude antibiotics [169]. Synergistic combinations with conventional antibiotics (Figure 3) offer an effective approach to counter resistance by simultaneously disrupting multiple genetic and metabolic pathways, thereby impairing bacterial defense mechanisms and compromising cellular integrity [166]. Clinically, the synergy with conventional antibiotics represents a major translational advantage, for instance, co-administration of silver or zinc-based VC-MNPs with vancomycin or β-lactams has restored antibiotic sensitivity in resistant strains [170,171]. However, the primary challenge lies in maintaining biocompatibility and preventing unintended microbiome disruption, which demands rigorous dose optimization and long-term toxicity evaluation [172,173].

### 5.2. Anti-Cancer Mechanisms

#### 5.2.1. Targeted Delivery to Tumors

VC-MNPs offer exceptional opportunities for targeted cancer therapy through exploitation of the enhanced vitamin requirements of rapidly dividing cancer cells. Malignant tumors exhibit increased demand for essential vitamins, resulting in overexpression of vitamin receptors on cancer cell surfaces compared to normal cells. This differential expression provides a molecular basis for selective targeting that can be exploited for therapeutic advantage. Folate-AuNPs bind folate receptors highly expressed in epithelial cancers, enhancing tumor selectivity while reducing systemic exposure. Similarly, cobalamin (B12)-conjugated NPs leverage the upregulated transcobalamin receptor system in rapidly proliferating tumors, achieving selective intracellular delivery [174]. Meanwhile, vitamin D3-based nanoplatforms offer dual functionality by serving both as targeting ligands and as bioactive molecules that regulate calcium signaling and promote cellular differentiation, highlighting their potential in cancer therapy and regenerative medicine [175,176,177]. Their targeting efficiency is reinforced by the enhanced permeability and retention (EPR) effect and pH-responsive release mechanisms, ensuring controlled drug liberation in acidic tumor microenvironments (pH 5.5–6.5). This pH-triggered release ensures that therapeutic agents are preferentially activated within the target tissue [176,177,178].

#### 5.2.2. Induction of Apoptosis in Cancer Cells

VC-MNPs trigger apoptosis through DNA damage, mitochondrial dysfunction, and lysosomal destabilization [179,180]. The anticancer mechanisms of VC-MNPs are summarized in Figure 3. Studies have demonstrated that dual drug-loaded vitamin D3 NPs containing cisplatin or doxorubicin with PI103 kinase inhibitor sustain intracellular exposure, amplifying DNA fragmentation and activating caspase-mediated death pathways [181]. The nanoparticle delivery system ensures sustained release of DNA damaging agents directly within cancer cells, maximizing therapeutic efficacy while minimizing systemic toxicity [176]. Multi-target apoptotic signaling is achieved through the simultaneous delivery of multiple therapeutic agents. In addition to direct DNA-targeted mechanisms, mitochondrial pathway activation significantly contributes to the pro-apoptotic effects of vitamin-conjugated nanoparticles. For example, vitamin C-conjugated NPs generate reactive oxygen species (ROS) and disrupt the redox balance of cancer cells. Interestingly, these effects are concentration-dependent, such that low concentrations exhibit protective antioxidant effects, while higher doses induce oxidative cell death. This selectivity is particularly effective in tumor cells, which are inherently more vulnerable to oxidative stress due to altered metabolic states [61]. In addition to mitochondrial dysfunction, lysosomal involvement also contributes to the cytotoxic responses. Endocytic uptake followed by lysosomal accumulation leads to pH-triggered release of cytotoxic payloads and membrane permeabilization, releasing apoptogenic enzymes into the cytosol [182].

#### 5.2.3. Mechanisms of Drug Resistance Modulation

Cancer drug resistance remains a major obstacle to successful chemotherapy, but vitamin-conjugated nanoparticles offer several mechanisms to overcome and modulate resistance in cancer cells. MDR circumvention is achieved through nanoparticle-mediated drug delivery that bypasses efflux pump mechanisms [183]. Traditional anticancer agents are rapidly expelled from tumor cells via ATP-binding cassette (ABC) transporters, which are upregulated in MDR phenotypes, leading to inadequate intracellular drug accumulation. In contrast, vitamin-conjugated nanoparticles can bypass these transporter-mediated efflux mechanisms by entering cells through receptor-mediated endocytosis, a route that avoids the classical efflux pumps [176]. Combination therapy approaches using vitamin-conjugated nanoparticles have demonstrated superior efficacy against drug-resistant cancer cell lines such as HER2-positive breast cancer, non-small cell lung cancer, and ovarian cancer, where synergistic effects with chemotherapeutics like paclitaxel, gemcitabine, and doxorubicin have restored drug sensitivity and enhanced tumor suppression [184,185,186].

Vitamin D_3_–PI103–proflavine NPs undergo rapid endocytic uptake in resistant cancer cells, facilitated by changes in membrane composition and elevated metabolic activity [176,181]. After internalization, their controlled release sustains intracellular drug levels, impairing efflux-based defense systems. Simultaneous vitamin-receptor engagement and co-delivery of chemotherapeutics modulate the PI3K/Akt and apoptotic signaling cascades, intensifying cellular stress. This integrated mechanism collectively disrupts resistance pathways and strengthens overall anticancer efficacy. Moreover, VC-MNPs modulate the tumor microenvironment, improving oxygenation, buffering acidity, and down regulating inflammatory cytokines that sustain resistance phenotypes [185,186]. These microenvironmental adjustments enhance penetration and reduce hypoxia-driven drug exclusion, positioning VC-MNPs as next-generation tools for MDR management [187].

### 5.3. Influence of Vitamin Functionalization

Functionalizing MNPs with vitamins has emerged as a promising strategy to enhance performance in biomedical applications. This approach leverages the natural affinity of vitamins for specific cellular receptors, improving both targeting efficiency and safety profiles of nanoparticle-based systems. By integrating vitamins into nanocarrier platforms, researchers have achieved significant advances in controlled delivery, biocompatibility, and therapeutic performance [188,189].

#### 5.3.1. Enhanced Cellular Uptake

Vitamin functionalization, particularly through nanoengineering and encapsulation strategies, has significantly enhanced the cellular uptake of vitamins. By modifying vitamins as part of nanocarrier systems such as NPs, micelles, or nanoliposomes their stability and solubility are improved, which allows for more efficient transport across cell membranes and targeted delivery within biological systems [34,190]. For example, B12-modified NPs bypass intestinal efflux via clathrin-mediated uptake [191]. Similarly, vitamin A-modified polymer micelles demonstrate targeted uptake by hepatic stellate cells, enhancing delivery specificity and cellular internalization in liver tissue [192]. Chitosan-coated NPs encapsulating vitamin B2 also show significantly higher uptake in intestinal epithelial cells, indicating improved transport across biological barriers [193]. Additionally, co-formulation with lyso-phosphatidylcholine further enhances membrane permeability and intracellular vitamin accumulation [194]. Together, these mechanisms improve absorption, stability, and bioavailability which are key parameters for translation from in vitro systems to clinical applications [34].

#### 5.3.2. Improved Biocompatibility and Efficacy

Targeted delivery combined with reduced toxicity results in better therapeutic outcomes. Magnetic NPS functionalized with vitamins have demonstrated significant potential across various biomedical fields, such as drug delivery, photothermal therapy, and gene transfection [195]. Their capability to accumulate selectively in diseased tissues enables higher concentrations of therapeutics locally while limiting systemic side effects [125,196]. Their surface chemistry supports cell proliferation, reduces immune activation, and allows controlled release of active compounds [197,198,199]. For example, In vivo, vitamin A-modified micelles suppress fibrotic markers without inflammation, while B12-functionalized magnetic NPs enable safe gene delivery and photothermal therapy [183,200,201]. In addition to surface modification, encapsulating vitamins within biocompatible carriers, such as chitosan-coated NPs, enhances stability, prolongs release, and protects against degradation, leading to more effective supplementation and therapeutic efficacy. These strategies collectively demonstrate that vitamin functionalization can optimize both the safety and performance of drug delivery systems, making them more suitable for clinical and nutraceutical applications [125]. Ongoing studies into vitamin–nanoparticle conjugates are expected to uncover innovative nanomedicine strategies with strong clinical potential [202].

## 6. Challenges and Limitations

VC-MNPs represent a promising approach for enhancing bioavailability and therapeutic efficacy, yet several significant challenges impede their clinical translation. These limitations span stability concerns, toxicity issues, regulatory hurdles, patient specific variability, and manufacturing scalability, each requiring careful consideration for successful development and implementation. Among these challenges, stability is particularly critical, as the interaction of vitamin-functionalized metallic nanoparticles with complex biological environments introduces multiple barriers that can undermine their performance [203]. One of the primary concerns is the formation of a protein corona that can mask vitamin ligands, diminishing receptor targeting and altering pharmacokinetics [204]. Additionally, fluctuations in pH and ionic strength across different physiological compartments can induce nanoparticle aggregation or premature degradation, while enzymatic activity can cleave vitamin–nanoparticle linkages, leading to uncontrolled release or loss of functionality [116,205]. Taken together, these factors underscore the complexity of achieving stable, reliable, and clinically translatable vitamin-conjugated metal nanoparticle systems.

Despite their biomedical promise in improving site-specific delivery and therapeutic efficacy, VC-MNPs raise substantial concerns regarding biocompatibility and systemic safety. While surface functionalization with vitamins is intended to enhance cellular uptake and targeting, the intrinsic properties of certain metallic cores such as silver or cadmium pose risks due to ion leaching, which can trigger oxidative stress, mitochondrial dysfunction [206], and inflammatory responses in host tissues [207,208]. Furthermore, the long-term accumulation of non-biodegradable NPs in clearance organs such as the liver, spleen, and kidneys exacerbate the potential for chronic toxicity, thereby undermining the advantages of targeted delivery [209,210,211]. Immunogenicity is another critical issue, as surface modifications may inadvertently activate immune pathways, leading to hypersensitivity or off-target effects [212]. Quantitative assessment of toxicity thresholds, biodistribution profiles, and clearance kinetics is still limited, underscoring the need for systematic in vivo and pharmacokinetic modeling studies [213]. Compared with lipid or polymer based nanocarriers which are biodegradable VC-MNPs face distinct challenges arising from their metallic cores, slower elimination rates, and greater potential for dose dependent oxidative injury [214,215].

The clinical translation of VC-MNPs is impeded by a lack of standardized protocols for synthesis, characterization, and quality control. Regulatory agencies such as the US Food and Drug Administration (FDA), and the European Medicine Agency (EMA) require comprehensive data on pharmacodynamics, toxicology, and long-term safety, which are often difficult to obtain due to the complex and heterogeneous nature of nanoparticle formulations. Furthermore, the ambiguous classification of VC-MNPs straddling the domains of pharmaceuticals, biologics, and medical devices complicates the regulatory approval process and necessitates multidisciplinary evaluation frameworks [216]. However, nanomedicine lacks universal characterization standards, complicating comparisons between studies and delaying approval. Moreover, establishing validated manufacturing and analytical protocols is essential for compliance with Good Manufacturing Practice (GMP) frameworks and for achieving reproducible product quality [217,218].

Scaling up the production of VC-MNPs from laboratory to industrial levels presents formidable technical and economic challenges. Reproducibility is a major concern, as maintaining uniformity in particle size, surface charge, and ligand density is difficult under large-scale synthesis conditions. While green and biologically inspired synthesis methods offer environmental advantages, they often lack the precision and throughput required for commercial manufacturing. Additionally, downstream purification processes to remove unbound ligands, reaction byproducts, and residual solvents are labor-intensive and may compromise nanoparticle stability or functionality [219,220].

Economic feasibility also depends on sustainable sourcing of vitamins and metals, cost-efficient purification and packaging systems, and integration into existing pharmaceutical supply chains. Addressing these aspects through process optimization, automation, and scalable continuous-flow synthesis will be crucial for commercialization [221,222]. Patient specific factors including metabolism, receptor expression, and immune status profoundly affect nanoparticle uptake, distribution, and clearance. Variability in folate or cobalamin receptor density can alter targeting efficiency, while metabolic or immune abnormalities influence therapeutic outcomes. Integrating pharmacogenomics with computational and machine-learning models will be essential to predict host–nanoparticle interactions, personalize dosing, and minimize adverse effects [223].

## 7. Future Perspectives

The future trajectory of vitamin-conjugated nanoparticles is expected to be shaped by significant advancements in synthesis methodologies, particularly through the creation of innovative conjugates that utilize bio-responsive linkers and precision-controlled fabrication techniques. These developments are enhancing the structural uniformity and functional specificity of NPs at the atomic scale [39]. Beyond structural considerations, deeper understanding of underlying mechanisms at molecular and genomic levels is becoming increasingly important. In the short term, research should focus on antimicrobial coatings, targeted drug delivery, and biosensing applications supported by preclinical safety and pharmacokinetic studies. Long-term goals include multifunctional theranostic systems for neurological, metabolic, and immunological diseases, as well as gene or RNA delivery platforms [224,225].

Despite these advances, several limitations remain to be addressed. Concerns regarding nanoparticle stability in complex biological and food matrices, potential off-target genetic or molecular effects, long-term toxicity, and environmental accumulation require careful evaluation. Overcoming scale-up challenges will require continuous-flow synthesis, modular click-linker chemistry, and inline quality control to ensure batch reproducibility and cost-effective production. Mechanistic studies using omics and long-term toxicity models will refine design rules and support regulatory alignment with FDA and EMA standards [226]. Nevertheless, this progress is being accelerated by interdisciplinary collaborations that bridge chemistry, biology, and clinical sciences, fostering a systems-level approach to nanoparticle design and deployment [227]. As these technologies evolve, increasing attention is being directed toward their clinical translation, with a focus on scalable production, regulatory alignment, and validation through clinical trials-key steps toward achieving commercial viability and integration into routine medical practice [190,228].

## 8. Conclusions

VC-MNPs represent an innovative generation of multifunctional nanocarriers that uniquely combine the therapeutic activity of vitamins with the structural precision and physicochemical tunability of metal cores. Unlike conventional lipid- or polymer-based nanocarriers, which primarily serve as passive delivery vehicles, VC-MNPs exhibit dual functionality simultaneously acting as bioactive agents and targeted delivery systems. This integrated approach enhances cellular uptake, enables receptor-mediated targeting, and amplifies therapeutic efficacy while minimizing off-target toxicity. Key insights from recent studies highlight their ability to overcome drug resistance, reduce systemic toxicity, and synergize with existing therapies. Despite considerable progress, challenges such as stability in biological environments, safety profiles, regulatory approval, and large-scale production must be addressed for successful clinical translation. Moreover, the lack of standardized synthesis protocols and limited scalability hinder their clinical translation.

Future research should focus on developing robust, reproducible synthesis methods, exploring novel vitamin-metal combinations, and conducting comprehensive in vivo studies to evaluate safety and efficacy. Interdisciplinary collaboration will be essential to bridge the gap between laboratory innovation and clinical application. Ultimately, VC-MNPs hold transformative potential in precision medicine, offering a pathway toward more effective, personalized, and less invasive therapeutic strategies. Therefore, VC-MNPs represent a new frontier in nanomedicine, uniting therapeutic and targeting functions beyond conventional nanocarriers. Continued innovation in design, safety, and scalability could enable these hybrids to advance precision medicine through safer, smarter, and more effective treatments for infectious and malignant diseases.

## Data Availability

The supporting data used in this manuscript has been obtained by Web of Science, Google Scholar, PubMed and Scopus.

## References

[1] Bayda S., Adeel M., Tuccinardi T., Cordani M., Rizzolio F. (2019). The History of Nanoscience and Nanotechnology: From Chemical–Physical Applications to Nanomedicine. Molecules.

[2] Maghsoudnia N., Eftekhari R.B., Sohi A.N., Zamzami A., Dorkoosh F.A. (2020). Application of Nano-Based Systems for Drug Delivery and Targeting: A Review. J. Nanopart. Res..

[3] Mu W., Chu Q., Liu Y., Zhang N. (2020). A Review on Nano-Based Drug Delivery System for Cancer Chemoimmunotherapy. Nano-Micro Lett..

[4] Kulkarni J.A., Witzigmann D., Thomson S.B., Chen S., Leavitt B.R., Cullis P.R., Van Der Meel R. (2021). The Current Landscape of Nucleic Acid Therapeutics. Nat. Nanotechnol..

[5] Rahman M.A., Jalouli M., Yadab M.K., Al-Zharani M. (2025). Progress in Drug Delivery Systems Based on Nanoparticles for Improved Glioblastoma Therapy: Addressing Challenges and Investigating Opportunities. Cancers.

[6] Lu B., Lv X., Le Y. (2019). Chitosan-Modified PLGA Nanoparticles for Control-Released Drug Delivery. Polymers.

[7] Qin Y., Ou L., Zha L., Zeng Y., Li L. (2023). Delivery of Nucleic Acids Using Nanomaterials. Mol. Biomed..

[8] Yaqoob A.A., Ahmad H., Parveen T., Ahmad A., Oves M., Ismail I.M.I., Qari H.A., Umar K., Mohamad Ibrahim M.N. (2020). Recent Advances in Metal Decorated Nanomaterials and Their Various Biological Applications: A Review. Front. Chem..

[9] Janjua T.I., Cao Y., Yu C., Popat A. (2021). Clinical Translation of Silica Nanoparticles. Nat. Rev. Mater..

[10] Jiang Y., Zhou Y., Zhang C.Y., Fang T. (2020). Co-Delivery of Paclitaxel and Doxorubicin by pH-Responsive Prodrug Micelles for Cancer Therapy. Int. J. Nanomed..

[11] Ibrahim Khan K.S., Khan I. (2019). Nanoparticles: Properties, Applications and Toxicities. Arab. J. Chem..

[12] Rabaan A.A., Bukhamsin R., AlSaihati H., Alshamrani S.A., AlSihati J., Al-Afghani H.M., Alsubki R.A., Abuzaid A.A., Al-Abdulhadi S., Aldawood Y. (2022). Recent Trends and Developments in Multifunctional Nanoparticles for Cancer Theranostics. Molecules.

[13] Meyers J. (2023). The Importance of Drug Delivery: An Innovative Approach for Revolutionizing Healthcare. Drug Des. Open Access.

[14] Karahmet Sher E., Alebić M., Marković Boras M., Boškailo E., Karahmet Farhat E., Karahmet A., Pavlović B., Sher F., Lekić L. (2024). Nanotechnology in Medicine Revolutionizing Drug Delivery for Cancer and Viral Infection Treatments. Int. J. Pharm..

[15] Kassaee S.N., Richard D., Ayoko G.A., Islam N. (2024). Lipid Polymer Hybrid Nanoparticles against Lung Cancer and Their Application as Inhalable Formulation. Nanomedicine.

[16] Liu S. (2024). Self-Assembled Lipid-Based Nanoparticles for Chemotherapy against Breast Cancer. Front. Bioeng. Biotechnol..

[17] Mojarad-Jabali S., Roh K.-H. (2025). Peptide-Based Inhibitors and Nanoparticles: Emerging Therapeutics for Alzheimer’s Disease. Int. J. Pharm..

[18] Lu D., Fan X. (2024). Insights into the Prospects of Nanobiomaterials in the Treatment of Cardiac Arrhythmia. J. Nanobiotechnol..

[19] Cheng X., Xie Q., Sun Y. (2023). Advances in Nanomaterial-Based Targeted Drug Delivery Systems. Front. Bioeng. Biotechnol..

[20] Prabahar K., Alanazi Z., Qushawy M. (2021). Targeted Drug Delivery System: Advantages, Carriers and Strategies. Indian J. Pharm. Educ..

[21] Wang X., Zhao X., Zhong Y., Shen J., An W. (2022). Biomimetic Exosomes: A New Generation of Drug Delivery System. Front. Bioeng. Biotechnol..

[22] Yetisgin A.A., Cetinel S., Zuvin M., Kosar A., Kutlu O. (2020). Therapeutic Nanoparticles and Their Targeted Delivery Applications. Molecules.

[23] Luo S., Lv Z., Yang Q., Chang R., Wu J. (2023). Research Progress on Stimulus-Responsive Polymer Nanocarriers for Cancer Treatment. Pharmaceutics.

[24] Thatyana M., Dube N.P., Kemboi D., Manicum A.-L.E., Mokgalaka-Fleischmann N.S., Tembu J.V. (2023). Advances in Phytonanotechnology: A Plant-Mediated Green Synthesis of Metal Nanoparticles Using Phyllanthus Plant Extracts and Their Antimicrobial and Anticancer Applications. Nanomaterials.

[25] Barker T. (2023). Vitamins and Human Health: Systematic Reviews and Original Research. Nutrients.

[26] Fagbohun O.F., Gillies C.R., Murphy K.P.J., Rupasinghe H.P.V. (2023). Role of Antioxidant Vitamins and Other Micronutrients on Regulations of Specific Genes and Signaling Pathways in the Prevention and Treatment of Cancer. Int. J. Mol. Sci..

[27] Sugandhi V.V., Pangeni R., Vora L.K., Poudel S., Nangare S., Jagwani S., Gadhave D., Qin C., Pandya A., Shah P. (2024). Pharmacokinetics of Vitamin Dosage Forms: A Complete Overview. Food Sci. Nutr..

[28] Shah S.T., Chowdhury Z.Z., Simarani K., Basirun W.J., Badruddin I.A., Hussien M., Alrobei H., Kamangar S. (2022). Nanoantioxidants: The Fourth Generation of Antioxidants—Recent Research Roadmap and Future Perspectives. Coatings.

[29] Bedhiafi T., Idoudi S., Fernandes Q., Al-Zaidan L., Uddin S., Dermime S., Billa N., Merhi M. (2023). Nano-Vitamin C: A Promising Candidate for Therapeutic Applications. Biomed. Pharmacother..

[30] Crintea A., Dutu A.G., Sovrea A., Constantin A.-M., Samasca G., Masalar A.L., Ifju B., Linga E., Neamti L., Tranca R.A. (2022). Nanocarriers for Drug Delivery: An Overview with Emphasis on Vitamin D and K Transportation. Nanomaterials.

[31] Fernández M., Javaid F., Chudasama V. (2018). Advances in Targeting the Folate Receptor in the Treatment/Imaging of Cancers. Chem. Sci..

[32] Fathi-karkan S., Sargazi S., Shojaei S., Farasati Far B., Mirinejad S., Cordani M., Khosravi A., Zarrabi A., Ghavami S. (2024). Biotin-Functionalized Nanoparticles: An Overview of Recent Trends in Cancer Detection. Nanoscale.

[33] Acharya C., Mishra S., Chaurasia S.K., Pandey B.K., Dhar R., Pandey J.K. (2025). Synthesis of Metallic Nanoparticles Using Biometabolites: Mechanisms and Applications. BioMetals.

[34] Aggeletopoulou I., Kalafateli M., Geramoutsos G., Triantos C. (2024). Recent Advances in the Use of Vitamin D Organic Nanocarriers for Drug Delivery. Biomolecules.

[35] Hsu C.-Y., Wang P.-W., Alalaiwe A., Lin Z.-C., Fang J.-Y. (2019). Use of Lipid Nanocarriers to Improve Oral Delivery of Vitamins. Nutrients.

[36] Karabasz A., Bzowska M., Szczepanowicz K. (2020). Biomedical Applications of Multifunctional Polymeric Nanocarriers: A Review of Current Literature. Int. J. Nanomed..

[37] Rezigue M., Krishnan A., Chuturgoon A. (2020). Lipid and Polymeric Nanoparticles: Drug Delivery Applications. Integrative Nanomedicine for New Therapies.

[38] Altammar K.A. (2023). A Review on Nanoparticles: Characteristics, Synthesis, Applications, and Challenges. Front. Microbiol..

[39] Kulkarni D., Sherkar R., Shirsathe C., Sonwane R., Varpe N., Shelke S., More M.P., Pardeshi S.R., Dhaneshwar G., Junnuthula V. (2023). Biofabrication of Nanoparticles: Sources, Synthesis, and Biomedical Applications. Front. Bioeng. Biotechnol..

[40] Zhang W., Taheri-Ledari R., Ganjali F., Afruzi F.H., Hajizadeh Z., Saeidirad M., Qazi F.S., Kashtiaray A., Sehat S.S., Hamblin M.R. (2022). Nanoscale Bioconjugates: A Review of the Structural Attributes of Drug-Loaded Nanocarrier Conjugates for Selective Cancer Therapy. Heliyon.

[41] Khan A., Rashid A., Younas R., Chong R. (2016). A Chemical Reduction Approach to the Synthesis of Copper Nanoparticles. Int. Nano Lett..

[42] Gutierrez F.V., Lima I.S., De Falco A., Ereias B.M., Baffa O., Diego de Abreu Lima C., Morais Sinimbu L.I., de la Presa P., Luz-Lima C., Damasceno Felix Araujo J.F. (2024). The Effect of Temperature on the Synthesis of Magnetite Nanoparticles by the Coprecipitation Method. Heliyon.

[43] Yadav A. (2024). A Review on Synthesis Methods of Materials Science and Nanotechnology. Adv. Mater. Lett..

[44] Daruich De Souza C., Ribeiro Nogueira B., Rostelato M.E.C.M. (2019). Review of the Methodologies Used in the Synthesis Gold Nanoparticles by Chemical Reduction. J. Alloys Compd..

[45] Iravani S., Korbekandi H., Mirmohammadi S.V., Zolfaghari B. (2014). Synthesis of Silver Nanoparticles: Chemical, Physical and Biological Methods. Res. Pharm. Sci..

[46] Villaverde-Cantizano G., Laurenti M., Rubio-Retama J., Contreras-Cáceres R., Mourdikoudis S. (2021). Reducing Agents in Colloidal Nanoparticle Synthesis—An Introduction. Reducing Agents in Colloidal Nanoparticle Synthesis.

[47] Banjara R.A., Kumar A., Aneshwari R.K., Satnami M.L., Sinha S.K. (2024). A Comparative Analysis of Chemical vs Green Synthesis of Nanoparticles and Their Various Applications. Environ. Nanotechnol. Monit. Manag..

[48] Gul M., Kashif M., Muhammad S., Azizi S., Sun H. (2025). Various Methods of Synthesis and Applications of Gold-Based Nanomaterials: A Detailed Review. Cryst. Growth Des..

[49] Jain K., Takuli A., Gupta T.K., Gupta D. (2024). Rethinking Nanoparticle Synthesis: A Sustainable Approach vs. Traditional Methods. Chem. Asian J..

[50] Reverberi A., Vocciante M., Lunghi E., Pietrelli L., Fabiano B. (2017). New Trends in the Synthesis of Nanoparticles by Green Methods. Chem. Eng. Trans..

[51] Antarnusa G., Jayanti P.D., Denny Y.R., Suherman A. (2022). Utilization of Co-Precipitation Method on Synthesis of Fe_3_O_4_/PEG with Different Concentrations of PEG for Biosensor Applications. Materialia.

[52] Kotresh M.G., Patil M.K., Inamdar S.R. (2021). Reaction Temperature Based Synthesis of ZnO Nanoparticles Using Co-Precipitation Method: Detailed Structural and Optical Characterization. Optik.

[53] Yazid N.A., Joon Y.C. (2019). Co-Precipitation Synthesis of Magnetic Nanoparticles for Efficient Removal of Heavy Metal from Synthetic Wastewater.

[54] Bokov D., Turki Jalil A., Chupradit S., Suksatan W., Javed Ansari M., Shewael I.H., Valiev G.H., Kianfar E. (2021). Nanomaterial by Sol-Gel Method: Synthesis and Application. Adv. Mater. Sci. Eng..

[55] Danks A.E., Hall S.R., Schnepp Z. (2016). The Evolution of ‘Sol–Gel’ Chemistry as a Technique for Materials Synthesis. Mater. Horiz..

[56] Nasr Azadani R., Sabbagh M., Salehi H., Cheshmi A., Beena Kumari A.R., Erabi G. (2021). Sol-Gel: Uncomplicated, Routine and Affordable Synthesis Procedure for Utilization of Composites in Drug Delivery: Review. J. Compos. Compd..

[57] Waqar M.A., Mubarak N., Khan A.M., Khan R., Khan I.N., Riaz M., Ahsan A., Munir M. (2024). Sol-Gel for Delivery of Molecules, Its Method of Preparation and Recent Applications in Medicine. Polym.-Plast. Technol. Mater..

[58] Gour A., Jain N.K. (2019). Advances in Green Synthesis of Nanoparticles. Artif. Cells Nanomed. Biotechnol..

[59] Singh H., Desimone M.F., Pandya S., Jasani S., George N., Adnan M., Aldarhami A., Bazaid A.S., Alderhami S.A. (2023). Revisiting the Green Synthesis of Nanoparticles: Uncovering Influences of Plant Extracts as Reducing Agents for Enhanced Synthesis Efficiency and Its Biomedical Applications. Int. J. Nanomed..

[60] Singh S., Mehra N.K., Jain N.K. (2016). Development and Characterization of the Paclitaxel Loaded Riboflavin and Thiamine Conjugated Carbon Nanotubes for Cancer Treatment. Pharm. Res..

[61] Chakraborty A., Jana N.R. (2017). Vitamin C-Conjugated Nanoparticle Protects Cells from Oxidative Stress at Low Doses but Induces Oxidative Stress and Cell Death at High Doses. ACS Appl. Mater. Interfaces.

[62] Nah H., Lee D., Heo M., Lee J.S., Lee S.J., Heo D.N., Seong J., Lim H.-N., Lee Y.-H., Moon H.-J. (2019). Vitamin D-Conjugated Gold Nanoparticles as Functional Carriers to Enhancing Osteogenic Differentiation. Sci. Technol. Adv. Mater..

[63] Han C., Nagendra V., Baig R., Varma R., Nadagouda M. (2015). Expeditious Synthesis of Noble Metal Nanoparticles Using Vitamin B12 under Microwave Irradiation. Appl. Sci..

[64] Malassis L., Dreyfus R., Murphy R.J., Hough L.A., Donnio B., Murray C.B. (2016). One-Step Green Synthesis of Gold and Silver Nanoparticles with Ascorbic Acid and Their Versatile Surface Post-Functionalization. RSC Adv..

[65] Ying S., Guan Z., Ofoegbu P.C., Clubb P., Rico C., He F., Hong J. (2022). Green Synthesis of Nanoparticles: Current Developments and Limitations. Environ. Technol. Innov..

[66] Xiao N., Li Y., Sun P., Zhu P., Wang H., Wu Y., Bai M., Li A., Ming W. (2024). A Comparative Review: Biological Safety and Sustainability of Metal Nanomaterials Without and with Machine Learning Assistance. Micromachines.

[67] Zain N.M., Stapley A.G.F., Shama G. (2014). Green Synthesis of Silver and Copper Nanoparticles Using Ascorbic Acid and Chitosan for Antimicrobial Applications. Carbohydr. Polym..

[68] El-Deeb N.M., Khattab S.M., Abu-Youssef M.A., Badr A.M.A. (2022). Green Synthesis of Novel Stable Biogenic Gold Nanoparticles for Breast Cancer Therapeutics via the Induction of Extrinsic and Intrinsic Pathways. Sci. Rep..

[69] Boopathi T.S., Rajiv A., Patel T.S.G.M., Bareja L., Salmen S.H., Aljawdah H.M., Arulselvan P., Suriyaprakash J., Thangavelu I. (2025). Efficient One-Pot Green Synthesis of Carboxymethyl Cellulose/Folic Acid Embedded Ultrafine CeO_2_ Nanocomposite and Its Superior Multi-Drug Resistant Antibacterial Activity and Anticancer Activity. Bioprocess Biosyst. Eng..

[70] Chen Z., Wu Y., Yao Z., Su J., Wang Z., Xia H., Liu S. (2022). 2D Copper(II) Metalated Metal–Organic Framework Nanocomplexes for Dual-Enhanced Photodynamic Therapy and Amplified Antitumor Immunity. ACS Appl. Mater. Interfaces.

[71] Haque S., Siddiqui S., Mashraqi A., Mandal R.K., Wahid M., Ahmad F., Fatima B. (2025). Green Synthesis and Biomedical Potential of L-Ascorbic Acid-Stabilized Copper Sulfide Nanoparticles as Antibacterial and Antioxidant Agents. Drug Dev. Ind. Pharm..

[72] Gu Y., Huang Y., Qiu Z., Xu Z., Li D., Chen L., Jiang J., Gao L. (2020). Vitamin B_2_ Functionalized Iron Oxide Nanozymes for Mouth Ulcer Healing. Sci. China Life Sci..

[73] Ghareghomi S., Ahmadian S., Zarghami N., Hemmati S. (2021). hTERT-Molecular Targeted Therapy of Ovarian Cancer Cells via Folate-Functionalized PLGA Nanoparticles Co-Loaded with MNPs/siRNA/Wortmannin. Life Sci..

[74] Song P., Wu Y., Fan M., Chen X., Dong M., Qiao W., Dong N., Wang Q. (2025). Folic Acid Modified Silver Nanoparticles Promote Endothelialization and Inhibit Calcification of Decellularized Heart Valves by Immunomodulation with Anti-Bacteria Property. Biomater. Adv..

[75] Safarpour R., Pooresmaeil M., Namazi H. (2024). Folic Acid Functionalized Ag@MOF(Ag) Decorated Carboxymethyl Starch Nanoparticles as a New Doxorubicin Delivery System with Inherent Antibacterial Activity. Int. J. Biol. Macromol..

[76] Pandey S.K., Singh S., Mehta S.K. (2018). Biocompatible Gadolinium Oxide Nanoparticles as Efficient Agent against Pathogenic Bacteria. J. Colloid Interface Sci..

[77] Rivas Aiello M.B., Ghilini F., Martínez Porcel J.E., Giovanetti L., Schilardi P.L., Mártire D.O. (2020). Riboflavin-Mediated Photooxidation of Gold Nanoparticles and Its Effect on the Inactivation of Bacteria. Langmuir.

[78] Kang X.-Q., Qiao Y., Lu X.-Y., Jiang S.-P., Li W.-S., Wang X.-J., Xu X.-L., Qi J., Xiao Y.-H., Du Y.-Z. (2019). Tocopherol Polyethylene Glycol Succinate-Modified Hollow Silver Nanoparticles for Combating Bacteria-Resistance. Biomater. Sci..

[79] Kandhasamy K., Chinnaiyan S.K., Balakrishnan K., Baskaran N., Ali S. (2025). Multifunctional Fucoidan and Folic Acid-Functionalized Yttrium Oxide Nanoparticles: A Novel Approach for Anti-Cancer, Antibacterial, Larvicidal and Environmental Remediation. Int. J. Biol. Macromol..

[80] Bužková A., Hochvaldová L., Večeřová R., Malina T., Petr M., Kašlík J., Kvítek L., Kolář M., Panáček A., Prucek R. (2025). Selenium Nanoparticles: Influence of Reducing Agents on Particle Stability and Antibacterial Activity at Biogenic Concentrations. Nanoscale.

[81] Moaness M., Mabrouk M., Ahmed M.M., Das D.B., Beherei H.H. (2022). Novel Zinc-Silver Nanocages for Drug Delivery and Wound Healing: Preparation, Characterization and Antimicrobial Activities. Int. J. Pharm..

[82] Zhou J., Zhang L., Wei Y., Wu Q., Mao K., Wang X., Cai J., Li X., Jiang Y. (2024). Photothermal Iron-Based Riboflavin Microneedles for the Treatment of Bacterial Keratitis via Ion Therapy and Immunomodulation. Adv. Healthc. Mater..

[83] Tudose M., Culita D.C., Ionita P., Chifiriuc M.C. (2015). Silver Nanoparticles Embedded into Silica Functionalized with Vitamins as Biological Active Materials. Ceram. Int..

[84] Maciejewska K., Marciniak L. (2022). Influence of the Synthesis Conditions on the Morphology and Thermometric Properties of the Lifetime-Based Luminescent Thermometers in YPO_4_: Yb^3+^, Nd^3+^ Nanocrystals. ACS Omega.

[85] Schiopu A.-G., Iordache D.M., Oproescu M., Cursaru L.M., Ioța A.-M. (2024). Tailoring the Synthesis Method of Metal Oxide Nanoparticles for Desired Properties. Crystals.

[86] Vijayaraghavan K., Ashokkumar T. (2017). Plant-Mediated Biosynthesis of Metallic Nanoparticles: A Review of Literature, Factors Affecting Synthesis, Characterization Techniques and Applications. J. Environ. Chem. Eng..

[87] Fernando I., Zhou Y. (2019). Impact of pH on the Stability, Dissolution and Aggregation Kinetics of Silver Nanoparticles. Chemosphere.

[88] Al Awadh A.A., Shet A.R., Patil L.R., Shaikh I.A., Alshahrani M.M., Nadaf R., Mahnashi M.H., Desai S.V., Muddapur U.M., Achappa S. (2022). Sustainable Synthesis and Characterization of Zinc Oxide Nanoparticles Using Raphanus Sativus Extract and Its Biomedical Applications. Crystals.

[89] Arya S., Mahajan P., Mahajan S., Khosla A., Datt R., Gupta V., Young S.-J., Oruganti S.K. (2021). Review—Influence of Processing Parameters to Control Morphology and Optical Properties of Sol-Gel Synthesized ZnO Nanoparticles. ECS J. Solid State Sci. Technol..

[90] Islam N.U., Jalil K., Shahid M., Rauf A., Muhammad N., Khan A., Shah M.R., Khan M.A. (2019). Green Synthesis and Biological Activities of Gold Nanoparticles Functionalized with Salix Alba. Arab. J. Chem..

[91] Ding H., Tan P., Fu S., Tian X., Zhang H., Ma X., Gu Z., Luo K. (2022). Preparation and Application of pH-Responsive Drug Delivery Systems. J. Control. Release.

[92] Payanda Konuk O., Alsuhile A.A.A.M., Yousefzadeh H., Ulker Z., Bozbag S.E., García-González C.A., Smirnova I., Erkey C. (2023). The Effect of Synthesis Conditions and Process Parameters on Aerogel Properties. Front. Chem..

[93] Bano A., Dawood A., Saira F., Malik A., Alkholief M., Ahmad H., Khan M.A., Ahmad Z., Bazighifan O. (2023). Enhancing Catalytic Activity of Gold Nanoparticles in a Standard Redox Reaction by Investigating the Impact of AuNPs Size, Temperature and Reductant Concentrations. Sci. Rep..

[94] Sánchez M.J.F., Sánchez M.D., Falcone R.D., Ritacco H.A. (2022). Production of Pd Nanoparticles in Microemulsions. Effect of Reaction Rates on the Particle Size. Phys. Chem. Chem. Phys..

[95] Yazdani S., Daneshkhah A., Diwate A., Patel H., Smith J., Reul O., Cheng R., Izadian A., Hajrasouliha A.R. (2021). Model for Gold Nanoparticle Synthesis: Effect of pH and Reaction Time. ACS Omega.

[96] Sharifi Dehsari H., Halda Ribeiro A., Ersöz B., Tremel W., Jakob G., Asadi K. (2017). Effect of Precursor Concentration on Size Evolution of Iron Oxide Nanoparticles. CrystEngComm.

[97] Carvalho P.M., Felício M.R., Santos N.C., Gonçalves S., Domingues M.M. (2018). Application of Light Scattering Techniques to Nanoparticle Characterization and Development. Front. Chem..

[98] Joudeh N., Linke D. (2022). Nanoparticle Classification, Physicochemical Properties, Characterization, and Applications: A Comprehensive Review for Biologists. J. Nanobiotechnol..

[99] Modena M.M., Rühle B., Burg T.P., Wuttke S. (2019). Nanoparticle Characterization: What to Measure?. Adv. Mater..

[100] Sarkar R., Rentenberger C., Rajagopalan J. (2015). Electron Beam Induced Artifacts During in Situ TEM Deformation of Nanostructured Metals. Sci. Rep..

[101] Xu X., Xia L., Zheng C., Liu Y., Yu D., Li J., Zhong S., Li C., Song H., Liu Y. (2025). Unravelling Nonclassical Beam Damage Mechanisms in Metal-Organic Frameworks by Low-Dose Electron Microscopy. Nat. Commun..

[102] Ma C., Arnold W. (2020). Nanoscale Ultrasonic Subsurface Imaging with Atomic Force Microscopy. J. Appl. Phys..

[103] Stiufiuc G.F., Stiufiuc R.I. (2024). Magnetic Nanoparticles: Synthesis, Characterization, and Their Use in Biomedical Field. Appl. Sci..

[104] Ngumbi P.K., Mugo S.W., Ngaruiya J.M. (2018). Determination of Gold Nanoparticles Sizes via Surface Plasmon Resonance. IOSR J Appl Chem.

[105] Dawadi S., Katuwal S., Gupta A., Lamichhane U., Thapa R., Jaisi S., Lamichhane G., Bhattarai D.P., Parajuli N. (2021). Current Research on Silver Nanoparticles: Synthesis, Characterization, and Applications. J. Nanomater..

[106] Prasad R.D., Prasad R.S., Prasad R.B., Prasad S.R., Singha S.B., Singha D., Prasad R.J., Sinha P., Saxena S., Vaidya A.K. (2024). A Review on Modern Characterization Techniques for Analysis of Nanomaterials and Biomaterials. ES Energy Environ..

[107] Wang X., Liu L.-H., Ramström O., Yan M. (2009). Engineering Nanomaterial Surfaces for Biomedical Applications. Exp. Biol. Med..

[108] Zhou X.-Q., Hayat Z., Zhang D.-D., Li M.-Y., Hu S., Wu Q., Cao Y.-F., Yuan Y. (2023). Zinc Oxide Nanoparticles: Synthesis, Characterization, Modification, and Applications in Food and Agriculture. Processes.

[109] Alharbi N.S., Alsubhi N.S., Felimban A.I. (2022). Green Synthesis of Silver Nanoparticles Using Medicinal Plants: Characterization and Application. J. Radiat. Res. Appl. Sci..

[110] Serrano-Lotina A., Portela R., Baeza P., Alcolea-Rodríguez V., Villarroel M., Ávila P. (2023). Zeta Potential as a Tool for Functional Materials Development. Catal. Today.

[111] Wahab S., Salman A., Khan Z., Khan S., Krishnaraj C., Yun S.-I. (2023). Metallic Nanoparticles: A Promising Arsenal against Antimicrobial Resistance—Unraveling Mechanisms and Enhancing Medication Efficacy. Int. J. Mol. Sci..

[112] Kamer A.M.A., El Maghraby G.M., Shafik M.M., Al-Madboly L.A. (2024). Silver Nanoparticle with Potential Antimicrobial and Antibiofilm Efficiency against Multiple Drug Resistant, Extensive Drug Resistant Pseudomonas Aeruginosa Clinical Isolates. BMC Microbiol..

[113] Patel J., Kumar G.S., Roy H., Maddiboyina B., Leporatti S., Bohara R.A. (2024). From Nature to Nanomedicine: Bioengineered Metallic Nanoparticles Bridge the Gap for Medical Applications. Discov. Nano.

[114] Shaikh S., Nazam N., Rizvi S.M.D., Ahmad K., Baig M.H., Lee E.J., Choi I. (2019). Mechanistic Insights into the Antimicrobial Actions of Metallic Nanoparticles and Their Implications for Multidrug Resistance. Int. J. Mol. Sci..

[115] Agreles M.A.A., Cavalcanti I.D.L., Cavalcanti I.M.F. (2022). Synergism between Metallic Nanoparticles and Antibiotics. Appl. Microbiol. Biotechnol..

[116] Ribeiro A.I., Dias A.M., Zille A. (2022). Synergistic Effects Between Metal Nanoparticles and Commercial Antimicrobial Agents: A Review. ACS Appl. Nano Mater..

[117] Sánchez-López E., Gomes D., Esteruelas G., Bonilla L., Lopez-Machado A.L., Galindo R., Cano A., Espina M., Ettcheto M., Camins A. (2020). Metal-Based Nanoparticles as Antimicrobial Agents: An Overview. Nanomaterials.

[118] Roy M., Hussain F. (2024). Mitigation of Breast Cancer Cells’ Invasiveness via Down Regulation of ETV7, Hippo, and PI3K/mTOR Pathways by Vitamin D3 Gold-Nanoparticles. Int. J. Mol. Sci..

[119] Wang L., Lin D., Li M., Jiang Y., Yang Y., Wang H., Chu H., Ye J., Liu Y. (2025). Bioactive Metallic Nanoparticles for Synergistic Cancer Immunotherapy. Acta Pharm. Sin. B.

[120] Xu J.-J., Zhang W.-C., Guo Y.-W., Chen X.-Y., Zhang Y.-N. (2022). Metal Nanoparticles as a Promising Technology in Targeted Cancer Treatment. Drug Deliv..

[121] Evans E.R., Bugga P., Asthana V., Drezek R. (2018). Metallic Nanoparticles for Cancer Immunotherapy. Mater. Today.

[122] Tian H., Zhang T., Qin S., Huang Z., Zhou L., Shi J., Nice E.C., Xie N., Huang C., Shen Z. (2022). Enhancing the Therapeutic Efficacy of Nanoparticles for Cancer Treatment Using Versatile Targeted Strategies. J. Hematol. Oncol..

[123] Zelechowska-Matysiak K., Salvanou E.-A., Bouziotis P., Budlewski T., Bilewicz A., Majkowska-Pilip A. (2023). Improvement of the Effectiveness of HER2+ Cancer Therapy by Use of Doxorubicin and Trastuzumab Modified Radioactive Gold Nanoparticles. Mol. Pharm..

[124] Beik J., Khademi S., Attaran N., Sarkar S., Shakeri-Zadeh A., Ghaznavi H., Ghadiri H. (2017). A Nanotechnology-Based Strategy to Increase the Efficiency of Cancer Diagnosis and Therapy: Folate-Conjugated Gold Nanoparticles. Curr. Med. Chem..

[125] Oyewumi M.O., Yokel R.A., Jay M., Coakley T., Mumper R.J. (2004). Comparison of Cell Uptake, Biodistribution and Tumor Retention of Folate-Coated and PEG-Coated Gadolinium Nanoparticles in Tumor-Bearing Mice. J. Control. Release.

[126] Rivas Aiello M.B., Castrogiovanni D., Parisi J., Azcárate J.C., García Einschlag F.S., Gensch T., Bosio G.N., Mártire D.O. (2018). Photodynamic Therapy in HeLa Cells Incubated with Riboflavin and Pectin-coated Silver Nanoparticles. Photochem. Photobiol..

[127] Zhao J., Hua W., Xu G., Gou S. (2017). Biotinylated Platinum(IV) Complexes Designed to Target Cancer Cells. J. Inorg. Biochem..

[128] Liu B., Duan H., Liu Z., Liu Y., Chu H. (2024). DNA-Functionalized Metal or Metal-Containing Nanoparticles for Biological Applications. Dalton Trans..

[129] Liu G., Swierczewska M., Lee S., Chen X. (2010). Functional Nanoparticles for Molecular Imaging Guided Gene Delivery. Nano Today.

[130] Karnwal A., Kumar Sachan R.S., Devgon I., Devgon J., Pant G., Panchpuri M., Ahmad A., Alshammari M.B., Hossain K., Kumar G. (2024). Gold Nanoparticles in Nanobiotechnology: From Synthesis to Biosensing Applications. ACS Omega.

[131] Wang S., Hossain M.d.Z., Han T., Shinozuka K., Suzuki T., Kuwana A., Kobayashi H. (2020). Avidin–Biotin Technology in Gold Nanoparticle-Decorated Graphene Field Effect Transistors for Detection of Biotinylated Macromolecules with Ultrahigh Sensitivity and Specificity. ACS Omega.

[132] Mirabello V., Calatayud D.G., Arrowsmith R.L., Ge H., Pascu S.I. (2015). Metallic Nanoparticles as Synthetic Building Blocks for Cancer Diagnostics: From Materials Design to Molecular Imaging Applications. J. Mater. Chem. B.

[133] Zheltova V., Vlasova A., Bobrysheva N., Abdullin I., Semenov V., Osmolowsky M., Voznesenskiy M., Osmolovskaya O. (2020). Fe_3_O_4_@HAp Core–Shell Nanoparticles as MRI Contrast Agent: Synthesis, Characterization and Theoretical and Experimental Study of Shell Impact on Magnetic Properties. Appl. Surf. Sci..

[134] Luo D., Wang X., Burda C., Basilion J.P. (2021). Recent Development of Gold Nanoparticles as Contrast Agents for Cancer Diagnosis. Cancers.

[135] Siddique S., Chow J.C.L. (2022). Recent Advances in Functionalized Nanoparticles in Cancer Theranostics. Nanomaterials.

[136] Hamimed S., Jabberi M., Chatti A. (2022). Nanotechnology in Drug and Gene Delivery. Naunyn. Schmiedebergs Arch. Pharmacol..

[137] Pan X., Veroniaina H., Su N., Sha K., Jiang F., Wu Z., Qi X. (2021). Applications and Developments of Gene Therapy Drug Delivery Systems for Genetic Diseases. Asian J. Pharm. Sci..

[138] Sung Y., Kim S. (2019). Recent Advances in the Development of Gene Delivery Systems. Biomater. Res..

[139] Wang C., Pan C., Yong H., Wang F., Bo T., Zhao Y., Ma B., He W., Li M. (2023). Emerging Non-Viral Vectors for Gene Delivery. J. Nanobiotechnol..

[140] Davis R.L. (2020). Mechanism of Action and Target Identification: A Matter of Timing in Drug Discovery. iScience.

[141] Huang M.L.-H., Chiang S., Kalinowski D.S., Bae D.-H., Sahni S., Richardson D.R. (2019). The Role of the Antioxidant Response in Mitochondrial Dysfunction in Degenerative Diseases: Cross-talk between Antioxidant Defense, Autophagy, and Apoptosis. Oxid. Med. Cell. Longev..

[142] Attaallah R., Amine A. (2021). The Kinetic and Analytical Aspects of Enzyme Competitive Inhibition: Sensing of Tyrosinase Inhibitors. Biosensors.

[143] Ray P.D., Huang B.-W., Tsuji Y. (2012). Reactive Oxygen Species (ROS) Homeostasis and Redox Regulation in Cellular Signaling. Cell. Signal..

[144] Gusti A.M.T., Qusti S.Y., Alshammari E.M., Toraih E.A., Fawzy M.S. (2021). Antioxidants-Related Superoxide Dismutase (SOD), Catalase (CAT), Glutathione Peroxidase (GPX), Glutathione-S-Transferase (GST), and Nitric Oxide Synthase (NOS) Gene Variants Analysis in an Obese Population: A Preliminary Case-Control Study. Antioxidants.

[145] Le Gal K., Schmidt E.E., Sayin V.I. (2021). Cellular Redox Homeostasis. Antioxidants.

[146] Li B., Ming H., Qin S., Nice E.C., Dong J., Du Z., Huang C. (2025). Redox Regulation: Mechanisms, Biology and Therapeutic Targets in Diseases. Signal Transduct. Target. Ther..

[147] Halliwell B. (2024). Understanding Mechanisms of Antioxidant Action in Health and Disease. Nat. Rev. Mol. Cell Biol..

[148] Modi S.K., Gaur S., Sengupta M., Singh M.S. (2023). Mechanistic Insights into Nanoparticle Surface-Bacterial Membrane Interactions in Overcoming Antibiotic Resistance. Front. Microbiol..

[149] Variyathody H., Arthanari M., Murugan M., Kuppannan G. (2023). Evaluating the Antibacterial Profile of Copper Oxide Nanoparticles—Vitamin E (CuO NPs-Vit E) Complex against Multidrug-Resistant Escherichia Coli and Staphylococcus Aureus. J. Pure Appl. Microbiol..

[150] More P.R., Pandit S., Filippis A.D., Franci G., Mijakovic I., Galdiero M. (2023). Silver Nanoparticles: Bactericidal and Mechanistic Approach against Drug Resistant Pathogens. Microorganisms.

[151] Qing Y., Cheng L., Li R., Liu G., Zhang Y., Tang X., Wang J., Liu H., Qin Y. (2018). Potential Antibacterial Mechanism of Silver Nanoparticles and the Optimization of Orthopedic Implants by Advanced Modification Technologies. Int. J. Nanomed..

[152] Fernando S.S.N., Gunasekara T., Holton J. (2018). Antimicrobial Nanoparticles: Applications and Mechanisms of Action. Sri Lankan J. Infect. Dis..

[153] Mortimer M., Wang Y., Holden P.A. (2021). Molecular Mechanisms of Nanomaterial-Bacterial Interactions Revealed by Omics—The Role of Nanomaterial Effect Level. Front. Bioeng. Biotechnol..

[154] Aunins T.R., Erickson K.E., Chatterjee A. (2020). Transcriptome-Based Design of Antisense Inhibitors Potentiates Carbapenem Efficacy in CRE *Escherichia coli*. Proc. Natl. Acad. Sci. USA.

[155] Afrasiabi S., Partoazar A. (2024). Targeting Bacterial Biofilm-Related Genes with Nanoparticle-Based Strategies. Front. Microbiol..

[156] Girma A., Alamnie G., Bekele T., Mebratie G., Mekuye B., Abera B., Workineh D., Tabor A., Jufar D. (2024). Greensynthesised silver nanoparticles: Antibacterial activity and alternative mechanisms of action to combat multidrug-resistant bacterial pathogens: A systematic literature review. Glob. Coalit. Lang. Rights.

[157] Wang L., Hu C., Shao L. (2017). The Antimicrobial Activity of Nanoparticles: Present Situation and Prospects for the Future. Int. J. Nanomed..

[158] Stevens D., Charlton-Sevcik A.K., Braswell W.E., Sayes C.M. (2025). Evaluating the Antibacterial Potential of Distinct Size Populations of Stabilized Zinc Nanoparticles. ACS Appl. Mater. Interfaces.

[159] Slavin Y.N., Asnis J., Häfeli U.O., Bach H. (2017). Metal Nanoparticles: Understanding the Mechanisms behind Antibacterial Activity. J. Nanobiotechnol..

[160] Khairnar S.V., Das A., Oupický D., Sadykov M., Romanova S. (2025). Strategies to Overcome Antibiotic Resistance: Silver Nanoparticles and Vancomycin in Pathogen Eradication. RSC Pharm..

[161] Mamun M.M., Sorinolu A.J., Munir M., Vejerano E.P. (2021). Nanoantibiotics: Functions and Properties at the Nanoscale to Combat Antibiotic Resistance. Front. Chem..

[162] Dutt Y., Dhiman R., Singh T., Vibhuti A., Gupta A., Pandey R.P., Raj V.S., Chang C.-M., Priyadarshini A. (2022). The Association between Biofilm Formation and Antimicrobial Resistance with Possible Ingenious Bio-Remedial Approaches. Antibiotics.

[163] Saffari Natanzi A., Poudineh M., Karimi E., Khaledi A., Haddad Kashani H. (2025). Innovative Approaches to Combat Antibiotic Resistance: Integrating CRISPR/Cas9 and Nanoparticles against Biofilm-Driven Infections. BMC Med..

[164] Ezeh C.K., Dibua M.E.U. (2024). Anti-Biofilm, Drug Delivery and Cytotoxicity Properties of Dendrimers. ADMET DMPK.

[165] Aguilar C., Jiménez A., Silva A., Kaur N., Thangarasu P., Ramos J., Singh N. (2015). Organic-Inorganic Hybrid Nanoparticles for Bacterial Inhibition: Synthesis and Characterization of Doped and Undoped ONPs with Ag/Au NPs. Molecules.

[166] Xie Y., Liu H., Teng Z., Ma J., Liu G. (2025). Nanomaterial-enabled anti-biofilm strategies: New opportunities for treatment of bacterial infections. Nanoscale.

[167] Imad Abd-AlAziz S., Thalij K.M., Zakari M.G. (2023). Inhibitory Susceptibility of Synthetic Selenium Nanoparticles and Some Conjugate Nutritional Compounds in Inhibition of Some Bacterial Isolates Causing Food Poisoning. Tikrit J. Agric. Sci..

[168] Fadwa A.O., Alkoblan D.K., Mateen A., Albarag A.M. (2021). Synergistic Effects of Zinc Oxide Nanoparticles and Various Antibiotics Combination against Pseudomonas Aeruginosa Clinically Isolated Bacterial Strains. Saudi J. Biol. Sci..

[169] Válková L., Hochvaldová L.S., Mistrík M., Kolář M., Langová K., Kolářová H., Štefková B., Prucek R., Kvítek L., Panáček A. (2025). Revealing the Mechanism of Synergistic Antibacterial Effect of Silver Nanoparticles in Combination with Vancomycin against *Enterococcus* Species by Fluorescence Microscopy Visualization. J. Mater. Chem. B.

[170] Sanati S., Bakhti A., Mohammadipanah F. (2025). Long-Term Toxic Effects of Nanoparticles on Human Microbiota. J. Trace Elem. Med. Biol..

[171] Mondal S.K., Chakraborty S., Manna S., Mandal S.M. (2024). Antimicrobial Nanoparticles: Current Landscape and Future Challenges. RSC Pharm..

[172] Bhole R.P., Jadhav S., Zambare Y.B., Chikhale R.V., Bonde C.G. (2020). Vitamin-Anticancer Drug Conjugates: A New Era for Cancer Therapy. Istanb. J. Pharm..

[173] Seraphin G., Rieger S., Hewison M., Capobianco E., Lisse T.S. (2023). The Impact of Vitamin D on Cancer: A Mini Review. J. Steroid Biochem. Mol. Biol..

[174] Palvai S., Nagraj J., Mapara N., Chowdhury R., Basu S. (2014). Dual Drug Loaded Vitamin D3 Nanoparticle to Target Drug Resistance in Cancer. RSC Adv..

[175] Patil S., Gawali S., Patil S., Basu S. (2013). Synthesis, Characterization and in Vitro Evaluation of Novel Vitamin D3 Nanoparticles as a Versatile Platform for Drug Delivery in Cancer Therapy. J. Mater. Chem. B.

[176] Sun L., Liu H., Ye Y., Lei Y., Islam R., Tan S., Tong R., Miao Y.-B., Cai L. (2023). Smart Nanoparticles for Cancer Therapy. Signal Transduct. Target. Ther..

[177] Kim J., Shim M.K., Moon Y., Kim J., Cho H., Yun W.S., Shim N., Seong J.-K., Lee Y., Lim D.-K. (2024). Cancer Cell-Specific and pro-Apoptotic SMAC Peptide-Doxorubicin Conjugated Prodrug Encapsulated Aposomes for Synergistic Cancer Immunotherapy. J. Nanobiotechnol..

[178] Zhao Y., Lei Y., Ning H., Zhang Y., Chen G., Wang C., Wan Q., Guo S., Liu Q., Xie R. (2023). PGF_2A_ Facilitates Pathological Retinal Angiogenesis by Modulating Endothelial FOS-driven ELR^+^ CXC Chemokine Expression. EMBO Mol. Med..

[179] Fageria L., Pareek V., Dilip R.V., Bhargava A., Pasha S.S., Laskar I.R., Saini H., Dash S., Chowdhury R., Panwar J. (2017). Biosynthesized Protein-Capped Silver Nanoparticles Induce ROS-Dependent Proapoptotic Signals and Prosurvival Autophagy in Cancer Cells. ACS Omega.

[180] Zhu L., Hu J., Wu X., Zhang J., Xu X., Huang X., Tian B., Zhao C.-X., Du Y., Wu L. (2025). Programmed Enhancement of Endogenous Iron-Mediated Lysosomal Membrane Permeabilization for Tumor Ferroptosis/Pyroptosis Dual-Induction. Nat. Commun..

[181] Liu B.-Y., Wu C., He X.-Y., Zhuo R.-X., Cheng S.-X. (2016). Multi-Drug Loaded Vitamin E-TPGS Nanoparticles for Synergistic Drug Delivery to Overcome Drug Resistance in Tumor Treatment. Sci. Bull..

[182] Choi J.Y., Thapa R.K., Yong C.S., Kim J.O. (2016). Nanoparticle-Based Combination Drug Delivery Systems for Synergistic Cancer Treatment. J. Pharm. Investig..

[183] Kydd J., Jadia R., Velpurisiva P., Gad A., Paliwal S., Rai P. (2017). Targeting Strategies for the Combination Treatment of Cancer Using Drug Delivery Systems. Pharmaceutics.

[184] Shrestha B., Tang L., Romero G. (2019). Nanoparticles-Mediated Combination Therapies for Cancer Treatment. Adv. Ther..

[185] Huang Z., Zhang Y., Li H., Zhou Y., Zhang Q., Chen R., Jin T., Hu K., Li S., Wang Y. (2019). Vitamin D Promotes the Cisplatin Sensitivity of Oral Squamous Cell Carcinoma by Inhibiting LCN2-Modulated NF-κB Pathway Activation through RPS3. Cell Death Dis..

[186] Liu J., Huang J., Zhang L., Lei J. (2021). Multifunctional Metal–Organic Framework Heterostructures for Enhanced Cancer Therapy. Chem. Soc. Rev..

[187] Khamis A., Gül D., Wandrey M., Lu Q., Knauer S.K., Reinhardt C., Strieth S., Hagemann J., Stauber R.H. (2022). The Vitamin D Receptor–BIM Axis Overcomes Cisplatin Resistance in Head and Neck Cancer. Cancers.

[188] Khursheed R., Dua K., Vishwas S., Gulati M., Jha N.K., Aldhafeeri G.M., Alanazi F.G., Goh B.H., Gupta G., Paudel K.R. (2022). Biomedical Applications of Metallic Nanoparticles in Cancer: Current Status and Future Perspectives. Biomed. Pharmacother..

[189] Truong T.T., Mondal S., Doan V.H.M., Tak S., Choi J., Oh H., Nguyen T.D., Misra M., Lee B., Oh J. (2024). Precision-Engineered Metal and Metal-Oxide Nanoparticles for Biomedical Imaging and Healthcare Applications. Adv. Colloid Interface Sci..

[190] Miah M.S., Chy W.R., Ahmed T., Suchi M., Muhtady A., Ahmad S.N.U., Hossain M.A. (2025). Emerging Trends in Nanotechnologies for Vitamin Delivery: Innovation and Future Prospects. Nano Trends.

[191] Singh A., Yadagiri G., Javaid A., Sharma K.K., Verma A., Singh O.P., Sundar S., Mudavath S.L. (2022). Hijacking the Intrinsic Vitamin B_12_ Pathway for the Oral Delivery of Nanoparticles, Resulting in Enhanced in Vivo Anti-Leishmanial Activity. Biomater. Sci..

[192] Liu X.Y., Li D., Li T.Y., Wu Y.-L., Piao J.S., Piao M.G. (2022). Vitamin A—Modified Betulin Polymer Micelles with Hepatic Targeting Capability for Hepatic Fibrosis Protection. Eur. J. Pharm. Sci..

[193] Sathiensathaporn S., Solé-Porta A., Baowan D., Pissuwan D., Wongtrakoongate P., Roig A., Katewongsa K.P. (2025). Nanoencapsulation of Vitamin B_2_ Using Chitosan-modified Poly(Lactic-*co*-glycolic Acid) Nanoparticles: Synthesis, Characterization, and in Vitro Studies on Simulated Gastrointestinal Stability and Delivery. J. Food Sci..

[194] Kotake-Nara E., Komba S., Hase M. (2021). Uptake of Vitamins D2, D3, D4, D5, D6, and D7 Solubilized in Mixed Micelles by Human Intestinal Cells, Caco-2, an Enhancing Effect of Lysophosphatidylcholine on the Cellular Uptake, and Estimation of Vitamins D’ Biological Activities. Nutrients.

[195] Albukhaty S., Sulaiman G.M., Al-Karagoly H., Mohammed H.A., Hassan A.S., Alshammari A.A.A., Ahmad A.M., Madhi R., Almalki F.A., Khashan K.S. (2024). Iron Oxide Nanoparticles: The Versatility of the Magnetic and Functionalized Nanomaterials in Targeting Drugs, and Gene Deliveries with Effectual Magnetofection. J. Drug Deliv. Sci. Technol..

[196] Du X., Yang X., Zhang Y., Gao S., Liu S., Ji J., Zhai G. (2022). Transdermal Delivery System Based on Heparin-Modified Graphene Oxide for Deep Transportation, Tumor Microenvironment Regulation, and Immune Activation. Nano Today.

[197] Chen G., Zhao Y., Xu Y., Zhu C., Liu T., Wang K. (2020). Chitosan Nanoparticles for Oral Photothermally Enhanced Photodynamic Therapy of Colon Cancer. Int. J. Pharm..

[198] Kim H.S., Lee D.Y. (2018). Near-Infrared-Responsive Cancer Photothermal and Photodynamic Therapy Using Gold Nanoparticles. Polymers.

[199] Huang R.-Y., Liu Z.-H., Weng W.-H., Chang C.-W. (2021). Magnetic Nanocomplexes for Gene Delivery Applications. J. Mater. Chem. B.

[200] Fidaleo M., Tacconi S., Sbarigia C., Passeri D., Rossi M., Tata A.M., Dini L. (2021). Current Nanocarrier Strategies Improve Vitamin B12 Pharmacokinetics, Ameliorate Patients’ Lives, and Reduce Costs. Nanomaterials.

[201] Kuldyushev N.A., Simonenko S.Y., Goreninskii S.I., Pallaeva T.N., Zamyatnin A.A., Parodi A. (2025). From Nutrient to Nanocarrier: The Multifaceted Role of Vitamin B12 in Drug Delivery. Int. J. Mol. Sci..

[202] Zeng L., Huang L., Han G. (2022). Dye Doped Metal-Organic Frameworks for Enhanced Phototherapy. Adv. Drug Deliv. Rev..

[203] Wang X., Sun T., Zhu H., Han T., Wang J., Dai H. (2020). Roles of pH, Cation Valence, and Ionic Strength in the Stability and Aggregation Behavior of Zinc Oxide Nanoparticles. J. Environ. Manag..

[204] Monopoli M.P., Åberg C., Salvati A., Dawson K.A. (2012). Biomolecular Coronas Provide the Biological Identity of Nanosized Materials. Nat. Nanotechnol..

[205] Lau C.P., Abdul-Wahab M.F., Jaafar J., Chan G.F., Rashid N.A.A. (2016). Effect of pH and Biological Media on Polyvinylpyrrolidone-Capped Silver Nanoparticles.

[206] Misra S.K., Rosenholm J.M., Pathak K. (2023). Functionalized and Nonfunctionalized Nanosystems for Mitochondrial Drug Delivery with Metallic Nanoparticles. Molecules.

[207] Lin Z., Monteiro-Riviere N.A., Riviere J.E. (2015). Pharmacokinetics of Metallic Nanoparticles. WIREs Nanomed. Nanobiotechnol..

[208] Nikolova M.P., Joshi P.B., Chavali M.S. (2023). Updates on Biogenic Metallic and Metal Oxide Nanoparticles: Therapy, Drug Delivery and Cytotoxicity. Pharmaceutics.

[209] Huang X., Ma Y., Li Y., Han F., Lin W. (2021). Targeted Drug Delivery Systems for Kidney Diseases. Front. Bioeng. Biotechnol..

[210] Jakic K., Selc M., Razga F., Nemethova V., Mazancova P., Havel F., Sramek M., Zarska M., Proska J., Masanova V. (2024). Long-Term Accumulation, Biological Effects and Toxicity of BSA-Coated Gold Nanoparticles in the Mouse Liver, Spleen, and Kidneys. Int. J. Nanomed..

[211] Shanahan K., Coen D., Nafo W. (2025). Polymer-Based Nanoparticles for Cancer Theranostics: Advances, Challenges, and Future Perspectives. Explor. BioMat-X.

[212] Devi L., Kushwaha P., Ansari T.M., Kumar A., Rao A. (2024). Recent Trends in Biologically Synthesized Metal Nanoparticles and Their Biomedical Applications: A Review. Biol. Trace Elem. Res..

[213] Yuan D., He H., Wu Y., Fan J., Cao Y. (2019). Physiologically Based Pharmacokinetic Modeling of Nanoparticles. J. Pharm. Sci..

[214] Egbuna C., Parmar V.K., Jeevanandam J., Ezzat S.M., Patrick-Iwuanyanwu K.C., Adetunji C.O., Khan J., Onyeike E.N., Uche C.Z., Akram M. (2021). Toxicity of Nanoparticles in Biomedical Application: Nanotoxicology. J. Toxicol..

[215] Min Y., Suminda G.G.D., Heo Y., Kim M., Ghosh M., Son Y.-O. (2023). Metal-Based Nanoparticles and Their Relevant Consequences on Cytotoxicity Cascade and Induced Oxidative Stress. Antioxidants.

[216] Geigert J. (2023). The Challenge of CMC Regulatory Compliance for Biopharmaceuticals.

[217] Foulkes R., Man E., Thind J., Yeung S., Joy A., Hoskins C. (2020). The Regulation of Nanomaterials and Nanomedicines for Clinical Application: Current and Future Perspectives. Biomater. Sci..

[218] Souto E.B., Silva G.F., Dias-Ferreira J., Zielinska A., Ventura F., Durazzo A., Lucarini M., Novellino E., Santini A. (2020). Nanopharmaceutics: Part I—Clinical Trials Legislation and Good Manufacturing Practices (GMP) of Nanotherapeutics in the EU. Pharmaceutics.

[219] Chandrakala V., Aruna V., Angajala G. (2022). Review on Metal Nanoparticles as Nanocarriers: Current Challenges and Perspectives in Drug Delivery Systems. Emergent Mater..

[220] Feng J., Guo X., Ramlawi N., Pfeiffer T.V., Geutjens R., Basak S., Nirschl H., Biskos G., Zandbergen H.W., Schmidt-Ott A. (2016). Green Manufacturing of Metallic Nanoparticles: A Facile and Universal Approach to Scaling Up. J. Mater. Chem. A.

[221] Paliwal R., Babu R.J., Palakurthi S. (2014). Nanomedicine Scale-up Technologies: Feasibilities and Challenges. AAPS PharmSciTech.

[222] Hemmrich E., McNeil S. (2024). Strategic Aspects for the Commercialization of Nanomedicines. J. Control. Release.

[223] Alghamdi M.A., Fallica A.N., Virzì N., Kesharwani P., Pittalà V., Greish K. (2022). The Promise of Nanotechnology in Personalized Medicine. J. Pers. Med..

[224] Cameron S.J., Sheng J., Hosseinian F., Willmore W.G. (2022). Nanoparticle Effects on Stress Response Pathways and Nanoparticle–Protein Interactions. Int. J. Mol. Sci..

[225] Patel T.A., Kevadiya B.D., Bajwa N., Singh P.A., Zheng H., Kirabo A., Li Y.-L., Patel K.P. (2023). Role of Nanoparticle-Conjugates and Nanotheranostics in Abrogating Oxidative Stress and Ameliorating Neuroinflammation. Antioxidants.

[226] Onyeaka H., Passaretti P., Miri T., Al-Sharify Z.T. (2022). The Safety of Nanomaterials in Food Production and Packaging. Curr. Res. Food Sci..

[227] Gawne P.J., Ferreira M., Papaluca M., Grimm J., Decuzzi P. (2023). New Opportunities and Old Challenges in the Clinical Translation of Nanotheranostics. Nat. Rev. Mater..

[228] Peng C. (2024). Editorial: Nanomedicine Development and Clinical Translation. Front. Chem..

